# Neurophysiological approaches to exploring emotional responses to cosmetics: a systematic review of the literature

**DOI:** 10.3389/fnhum.2024.1443001

**Published:** 2024-10-22

**Authors:** Audrey Diwoux, Damien Gabriel, Marie-Héloïse Bardel, Youcef Ben Khalifa, Pierre-Édouard Billot

**Affiliations:** ^1^Beauty Research and Performance Department, CHANEL Parfums Beauté, Pantin, France; ^2^Université de Franche-Comté, INSERM, UMR 1322 LINC, Besançon, France; ^3^Centre d'Investigation Clinique, Inserm, CIC 1431, CHU, Besançon, France; ^4^Plateforme de Neuroimagerie Fonctionnelle et Neuromodulation Neuraxess, Besançon, France

**Keywords:** cosmetics, neurosciences, physiological activity, brain activity, positive emotion, skincare, makeup, perfume

## Abstract

**Introduction:**

This systematic review explores the use of neurophysiological measurements to study emotional responses to cosmetic products. The aim is to evaluate existing literature on these measurements in cosmetics, identify the main findings, highlight methodological challenges, and propose new guidelines for future research.

**Method:**

A systematic search focusing on neurophysiological measures to determine emotions induced by different cosmetic products was carried out in accordance with PRISMA guidelines.

**Results:**

A total of 33 articles identified with the EBSCO database met the inclusion criteria. In all, 10 different measurement tools were used in these articles to assess the emotional effects of cosmetic products.

**Discussion:**

This review emphasizes the complexity of interactions between cosmetics and emotional responses. It underscores the importance of future research with a more holistic approach that couples several physiological measurements. Among them, electrophysiological brain activity shows potential for enhancing understanding of emotional responses related to cosmetic products. Frontal asymmetry, particularly in the alpha frequency band, was often use and frequently linked to positive emotional states, although conflicting evidence exists. Additionally, cardiac activity, specifically the LF/HF ratio, emerges as a promising marker for differentiating between different cosmetic products. However, methodological heterogeneity, present challenges for replicability, generalizability, and complicate data interpretation.

## 1 Introduction

Cosmetics play an essential role in our daily lives, helping to cleanse, protect, moisturize, nourish, and preserve the beauty of skin, hair, nails, and other parts of the body (Messaraa et al., 2020). Whether for personal care, improving appearance, or maintaining good hygiene, their use is widespread throughout the world, by both men and women (Lascaratos et al., 2004). These products offer the possibility of temporarily modifying or enhancing various aspects of our appearance, such as looking younger (Russell et al., 2019) or giving an impression of better health (Nash et al., 2006). They can also enhance femininity and attractiveness (Courrèges et al., 2014; Kosmala et al., 2019; Mileva et al., 2016; Ueno et al., 2014).

However, cosmetics are not limited to their aesthetic use, and can also influence our emotions (Courrèges et al., 2021; Korichi et al., 2008). By applying lipstick or perfume, we not only modify our appearance, but also stimulate our internal emotional state. Indeed, improvements in appearance help, for example, to boost self-image, self-confidence (Mohammed et al., 2023) and self-esteem (Kathleen, 2014). Thus, understanding the interactions between cosmetics and the complex neurophysiological mechanisms that regulate our emotions opens the way to a wealth of research aimed at revealing the impact of these products on overall wellbeing.

For this reason, the link between cosmetics and their emotional impacts has attracted growing interest in the field of scientific research, leading to studies aimed at better understanding how these products modulate our emotional states. For example, aesthetic improvements have been linked to increased wellbeing (Korichi et al., 2008; Kosmala et al., 2019; Matsuoka et al., 2006) and the use of cosmetics is often associated with positive emotions such as relaxation and mood enhancement (Courrèges et al., 2021; Marahatta et al., 2021; Segot-Chicq et al., 2007; Zhang et al., 2020). However, all these findings are mainly based on subjective data collection methods such as questionnaires, surveys, or focus groups (Trautmann et al., 2017). Yet these measures are often influenced by contextual factors and individual biases, and may vary depending on the time of year or day (Bergstrom et al., 2014). Participants may also encounter difficulties in fully recalling their emotions, which can affect responses to questionnaires. Finally, different interpretations and answers may be given for cultural reasons.

Therefore, to gain a more accurate understanding, objective methods based on psychophysiological and neurophysiological measurements are needed. Indeed, these measurements are the result of unconscious mental processes that cannot be voluntarily controlled, making them non-falsifiable measurements (Ivonin et al., 2015). By combining several of these measurements with subjective questionnaires, it is possible to gather more comprehensive information about an individual's emotional responses. Neurophysiology can then serve as a bridge between the subjective and the objective, enabling a deeper understanding of the processes underlying emotional responses elicited by the application of cosmetics.

A whole range of neurophysiological tools exist to objectively assess an emotional state. Among the many non-invasive physiological methods, we can cite electrodermal activity, cardiac activity, or respiration for example (as reviews of physiological methods for measuring emotions see, for example: Cai et al., 2023; Chunawale and Bedekar, 2020; Dzedzickis et al., 2020). Electrocardiography (ECG) measures heart muscle activity, expressed in beats per minute (heart rate). From this activity, several variables can be calculated to study various physiological aspects, such as the dominance of sympathetic activity. Cardiac activity is interesting for assessing subjects' emotions, as it represents a good indicator of emotional valence and therefore positive emotions (Agrafioti et al., 2012; Hachenberger et al., 2023; Meier et al., 2020). Electrodermal activity corresponds to the electrical characteristics of the skin originating from the eccrine sweat glands that cause skin perspiration (Grapperon et al., 2012). Variables derived from this activity provide a reliable measure of emotional arousal (for a review, see: Luauté et al., 2018; Posada-Quintero and Chon, 2020). Finally, the measurement of respiration aims to calculate the number of cycles per minute (inspiration + expiration). Respiration is rarely utilized in studies of emotions, yet it exhibits modifications in response to emotional changes (for a review, see: Homma and Masaoka, 2008).

Regarding neurophysiological methods, the most common include electroencephalography (EEG), functional near-infrared spectroscopy (fNIRS), and functional magnetic resonance imaging (fMRI) (for a review of the neural correlates of positive emotions with these different measures, see: de Vries et al., 2023). These measures allow us to record brain activity to visualize information processing and therefore, in our context, emotions. In EEG, it has been recognized for many years that valence can be calculated using frontal alpha asymmetry (FAA) (Davidson, 1992) (For a review on alpha asymmetry, see: Allen et al., 2018; Allen and Kline, 2004). This frontal alpha asymmetry refers to the difference in alpha-type brain activity (8–12 Hz) between the left and right frontal hemispheres (Harmon-Jones and Gable, 2018). In this model, it has been shown in numerous studies that greater activity in the left prefrontal cortex reflects positive emotions and emotional approach processes (Davidson, 1992, 1993). There is also a method for measuring arousal. The latter is more recent and has not been validated in quite so much research but consists in calculating the ratio between alpha and beta waves in the prefrontal cortex (Ramirez et al., 2015).

In fNIRS, the distribution of brain activity between the right and left hemispheres in the prefrontal cortex is also used in the study of emotions (Ishikawa et al., 2014). Thus, studies using this method rely on laterality indices to define in which hemisphere brain activity is dominant. Indeed, most studies have revealed increased activity in the prefrontal cortex during emotional experience (physiological and behavioral response to an emotion-invoking event or stimulus) (Westgarth et al., 2021). Finally, fMRI is a recording technique that enables visualization of the brain's overall functioning and provides a complete cerebral map of the different areas activated. This technique has excellent spatial resolution, enabling precise localization of brain structures activated in different contexts.

All these techniques are already widely used to measure emotions in various fields of research. In psychology, for example, they are used for emotional recognition (Emilee and Shashi, 2019; Rattel et al., 2020) (for a review, see: Cai et al., 2023). In psychiatric research, these data are used to better understand the mechanisms underlying emotional disorders such as anxiety (Dziembowska et al., 2016; Rosebrock et al., 2017) or depression (Stange et al., 2017) (for an EEG/depression review see, for example: Aguiar Neto and Rosa, 2019). In the marketing sector, the analysis of consumers' physiological reactions is employed when faced with advertisements or products offered under different conditions (Bettiga et al., 2020; Laureanti et al., 2022; Schoen et al., 2018; Torrico et al., 2020) (for a review of physiological measurements used in neuromarketing, see: Alvino et al., 2020).

While psychophysiological and neuroimaging data sets are used in many fields, offering rich, multidimensional insights into human emotional responses, the use of these methods is more recent and less widespread in cosmetics. This has led to a lack of consistency in protocols in this emerging field. It is for this reason that this review proposes to take stock using physiological and neurophysiological data in the field of cosmetics. Highlighting the various works that have adopted this approach and compiling a comprehensive inventory will enable us to explore the relevance of different neuroimaging and psychophysiological methods for studying the link between cosmetics and emotions, as well as deepening our knowledge of these complex interactions and the possibility of measuring them adequately.

The main aim of this review is therefore to analyze the methods used, following the PRISMA guidelines (Moher et al., 2015), in order to find out what the current procedures are and their relevance for studying the link between cosmetics and emotions. In this way, we hope to contribute to a better understanding of how different measures might enable us to analyze how cosmetics can elicit, modulate, and generate our emotions.

## 2 Method

### 2.1 Objectives

The main objective of this review is to find out whether the neurophysiological methods used are relevant to the different specificities associated with cosmetics. Indeed, the use of cosmetics is a holistic experience involving all the senses. This may call into question the relevance of certain neuroscience investigative methods traditionally used to measure emotions. For this reason, we wanted to focus on the multisensory aspect of using cosmetics.

What is more, the emotions felt when using a cosmetic are likely to evolve over time, throughout the product's discovery, application, or even once it has been applied. We would then like to determine whether these investigative methods can measure this temporal dynamic. Finally, when applying a cosmetic in everyday life, this is usually done by the person applying it to themselves. It is therefore a voluntary motor activity that raises at least one question. This consists in knowing how to take account of artifacts and problems linked to participants' movements, and whether the methods of investigation and the design of the protocols make it possible to take them into account. Our aim is to understand whether the measures we use allow us to reliably estimate the emotions aroused by cosmetics, considering all the specificities of cosmetics.

### 2.2 Methods

To gather the literature on studies using objective methods to measure cosmetics-induced emotions, a systematic review was conducted and the results reported in accordance with PRISMA guidelines (Moher et al., 2015).

#### 2.2.1 Literature search

##### 2.2.1.1 Definition

For the purposes of this literature review, our definition of emotions is that of the Federal Food, Drug & Cosmetic Act (FD&C Act), which defines the term “cosmetic” in Section 201(i) as “articles intended to be rubbed, poured, sprinkled, or sprayed on, introduced into, or otherwise applied to the human body...for cleansing, beautifying, promoting attractiveness, or altering the appearance.”

Concerning emotions, we include basic (primary) emotions as well as mixed emotions. Our definition of emotions is that of Scherer (Scherer and Grandjean, 2008), who defines emotion as a sequence of state changes occurring in five organ systems (cognitive, psychophysiological, motor, motivational, subjective) in an interdependent and synchronized manner, in response to the evaluation of the relevance of an external or internal stimulus to a central interest for the organism.”

##### 2.2.1.2 Bibliographic database

To gather relevant articles, the search was carried out on EBSCO, which brings together several databases (STM source; Psychology and Behavioral Sciences Collection; APA psychinfo; APA PsycArticles; Biomedical Reference Collection; and MEDLINE Complete). Filters were applied to search only for articles in English or French, relating to adult human subjects over 18 years of age (English/French, +18 years, and Human).

##### 2.2.1.3 Search equation

The search strategy involved a combination of keywords related to positive emotions *(positive affect, emotional evaluation, happiness, wellbeing, well being, well-being, wellness, mood, emotion, pleasantness, relaxation, satisfaction*, and *preference)*, neurophysiological measurements *(physiological response, physiological activity, autonomic nervous system, electrodermal activity, skin conductance, cardiac activity, heart rate, blood pressure, respiration, brain activity, EEG, fNIRS, MRI, prefrontal cortex, brain activation, cortisol levels, body temperature, EMG)* and cosmetics *(cosmetics, makeup, facial cream, perfume, lipstick, skincare, fragrance* and *aroma*).

To combine these categories of keywords, the Boolean search operators AND and OR were used. In addition, these keywords were specifically searched for in the article abstracts section to narrow down our searches.

#### 2.2.2 Study selection

##### 2.2.2.1 Period

Due to the small number of articles available in the field, the search strategy focused on all published articles, without limiting the publication period. The last searches for this journal were carried out on May 2024.

##### 2.2.2.2 Inclusion criteria

To determine the eligibility of the different articles, the PICO recommendations (P-population, I-induction, C-comparison, O-outcomes) were used to develop the research question. The central question of the study was:

What is the relevance of the neurophysiological data collected (O) for the study of emotions (C) induced by different cosmetics (I) in healthy adults (P)?

Thus, articles were included if they met the following criteria: (1) Use of cosmetic products; (2) Healthy adult subjects (over 18 years of age); (3) Language of publication in English or French; (4) Use of physiological measurements (as mentioned in the keywords); (5) Methods aimed at analyzing emotional responses.

##### 2.2.2.3 Exclusion criteria

Articles were excluded when: (1) The analysis was not directly related to cosmetic products; (2) The study involved clinical samples or pathological subjects; (3) The analysis was conducted on animals or *in vitro*; (4) The data measured were subjective and did not involve physiological measurements; (5) The article involved invasive procedures such as cosmetic surgery; and (6) The article was a review, critique, book chapter, thesis, summary or pre-publication. The exclusion criteria were grouped into three categories (see Supplementary Table S1).

##### 2.2.2.4 Study selection process

To select relevant articles for this review, titles and abstracts were carefully examined by two reviewers (PEB and AD). Duplicates were then eliminated before the articles from the literature search were analyzed. All remaining references were imported into Rayyan, an online application for systematic reviews (Ouzzani et al., 2016). The articles were classified into three categories: Inclusion, Exclusion, and Maybe, according to the selection criteria stated above.

##### 2.2.2.5 Recovery process

Uncertainties and disagreements were resolved through discussions between the two reviewers (PEB and AD) and two other investigators (MHB and DG). Where there was insufficient information to determine eligibility, the papers were subjected to further review (full-text reading). All articles selected in this stage were retained for a final full-text reading to determine the eligibility of studies in the journal.

##### 2.2.2.6 Eligibility of studies

This final stage was used to select the articles to be included in the review. It was only after reading all the previously selected articles that the two reviewers were able to determine the eligibility of each study. Some articles omitted during the search could be identified thanks to the bibliographic references of the selected articles.

##### 2.2.2.7 Summary of search procedure

**Figure 1:**
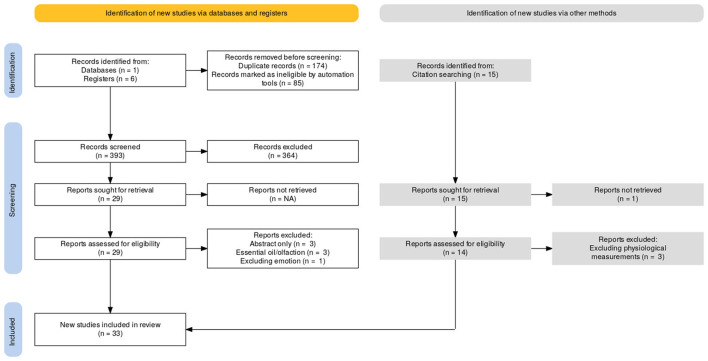
Overview of the search procedure leading to the final selection of 33 items.

A summary of the selection process is available in the PRISMA flow chart (see Figure 1).

The search equation resulted in a total of 652 items. After automatic and manual deletion of duplicates, 393 articles were retained for further processing. By scanning all titles and abstracts, a first selection was made, excluding articles that did not meet the inclusion criteria described above. Thus, 29 articles were retained for full reading. Of these 29 articles, seven were excluded for various reasons and 22 were selected for review. Additional articles were selected from the references of these articles. A snowball search was conducted from these citations. In this way, an additional 15 articles were included in the process. One of these was excluded after reading the abstract, as it was not relevant to our review, and three others were excluded after reading the full article. Thus, 11 additional articles met the eligibility criteria of the study. Thus, based on the full-text review, 33 articles met our selection criteria and were included in the review (22 articles from the search equation and 11 articles from search citations and a snowballing technique).

#### 2.2.3 Study analysis and data extraction

##### 2.2.3.1 Data extraction

For each study, the two reviewers (AD and PEB) extracted the following information: *Demographic data:* authors, title, years of publication, country of study, number of subjects, average age of subjects, gender of subjects, type of study; *Cosmetic data:* cosmetics used, brand of products, area and place of application, number and duration of applications, quantity of product used, mode of application, and sensory modality studied; *Physiological data:* physiological data recorded, method of analysis, control used, and time of recording; *Results:* physiological variations observed, whether or not products can be distinguished from each other, and main conclusions.

##### 2.2.3.2 Types of measurements extracted

Among the information selected, questionnaires and non-physiological measurements (facial expressions, ethograms, etc.) were not listed, as these were not within the scope of this review. Thus, the methods extracted in this review can be classified into two categories according to their nature: physiological tools and neurophysiological measurements.

These two types of measurement reflect the (largely unconscious) activity of the autonomic nervous system. Physiological tools include electrodermal activity (EDA), electrocardiogram (ECG), respiration, hormone/protein assays, and electromyography (EMG). Neurophysiological tools include electroencephalography (EEG), functional magnetic resonance imaging (fMRI), and functional near infrared spectroscopy (fNIRS). An overview of these techniques is given in Supplementary Table S2.

##### 2.2.3.3 Assessment of study quality

Quality assessment of eligible studies was also carried out independently by two investigators (PEB and AD) according to the criteria described. Two instruments developed by methodologists at the National Institute of Health to assess study quality were used (NHLBI, 2023). The quality assessment tool for observational cohort and cross-sectional studies (QATOCCS) was merged with the quality assessment tool for controlled intervention studies (QACIS). This merger was carried out to have an instrument more specific to the research of this review. The quality assessment grid for the studies used in this work can be found in Supplementary Table S3.

## 3 Results

Tables 1–4 present the data extracted for each study. Table 1 covers the main characteristics of the subjects and the study (sample, location, age mean, gender, type of study and quality of study). Table 2 includes cosmetic-related data (cosmetic used, product brand, application area, place: lab/home, number of applications, duration of stimulation, amount applied, mode of application, and sensory modality). Table 3 refers to the physiological measurements used (physiological data, data processing, temporal modality, artifact removal/filter, and comparison). Table 4 refers to study results (physiological results, distinction between products/conditions, and main conclusion).

**Table 1:** Main characteristics of subjects and studies.

**References**	**Research questions**	**Sample**	**Location**	**Age mean**	**Gender (F/M)**	**Type of study**	**Quality score**
Abriat et al. (2007)	Effects of perfume on mood and their short- and long-term physiological correlates (familiarization).	*N* = 37	France	(50–65 years)	F	Randomized controlled trial (RCT)	7/11
Baer et al. (2018)	Influence of contextual information evoking luxury on emotional responses to a perfume.	*N* = 21	Switzerland	22.7 ± 3.3	F	Randomized controlled trial (RCT)	6/11
Barkat et al. (2003)	Influence of cosmetic odor and color on autonomic responses.	*N* = 20	France	47 ± 7	F	Randomized controlled trial (RCT)	6/11
Boucsein et al. (2002)	Influence of different hair treatments on tactile perception.	*N* = 24	Germany	Not specified	M/F	Controlled trial	6/11
Bouhout et al. (2023)	Influence of a facial skincare on wellbeing.	*N* = 63	France	33	F	Controlled trial	5/11
Cabannes et al. (2019)	Comparison of the effects of two foundations before and after 5 days of application on psychophysiological parameters.	*N* = 40	France	35 ± 10	F	Randomized controlled trial (RCT) + single-blind, placebo, and crossover trial	8/11
Churchill and Behan (2010)	Comparison of three methods for studying emotions.	*N* = 22	UK	Not specified	Not specified	Blind + randomized trial	7/11
David et al. (2019)	Establish the positive emotions associated with a cosmetic cream.	*N* = 26	Romania	27.42	F	Randomized controlled trial (RCT)	7/11
Field et al. (2005)	Exploring the effects of the scent of lavender gel on relaxation.	*N* = 11	U.S. (Miami)	Not specified	Not specified	Not specified	6/11
Gabriel et al. (2021)	Compare the emotions induced by the application of two creams.	*N* = 15	France	(18–65 years)	F	Crossover study + pilot study	10/11
Hirabayashi et al. (2021)	Replicate a previous study and see if there is a link between activation of the prefrontal cortex and cosmetic appreciation.	*N* = 25	Japan	29.6	F	Randomized controlled trial (RCT)	7/11
Kawabata Duncan et al. (2019)	Assessing the links between brain activity and willingness to pay.	*N* = 30	Japan	21.2	F	Randomized controlled trial (RCT)	8/11
Kikuchi et al. (2021)	Establishing the brain areas involved in cosmetic attachment.	*N* = 20	Japan	33.4 ± 3.5	F	Randomized controlled trial (RCT)	7/11
Kim et al. (2022)	Using an algorithm to classify emotions.	*N* = 19	Korea	34.1	F	Randomized trial	6/11
Kokubo and Kawano (2016)	Measuring relaxation induced by two creams with different formulations.	*N* = 16	Japan	41.85	F	Randomized controlled trial (RCT)	7/11
Leão et al. (2017)	Evaluating the impact of cosmetic on stress.	*N* = 96	Brazil	(18–60 years)	F	Randomized controlled trial (RCT)	10/11
Leong (2022)	Compare the emotional responses of two products using two techniques.	*N* = 10	Singapore	Not specified	F	Not specified	3/11
Lombardi (2017)	Comparison of emotions felt after applying different products.	*N* = 20	France	35 ± 10	F	Randomized trial	6/11
Nagai et al. (2012)	Study the relationship between brain activity and cosmetic application.	*N* = 60	Japan (different nationalities)	30.6	F	Controlled trial	6/11
Ohira and Hirao (2015)	Evaluate preference between four products using electrodermal activity.	*N* = 33	Japan	40.7	F	Randomized trial	6/11
Painchault et al. (2020)	Evaluating the relaxing effects of cosmetics fragrances.	*N* = 55	Netherlands	Shampoo: 41.1; serum: 40.6	F	Randomized controlled trial (RCT)	7/11
Pichon et al. (2015)	Use physiology to study olfactory responses associated with fragrances.	*N* = 21	Switzerland	22.7 ± 3.3	F	Randomized study	7/11
Pössel et al. (2005)	Influence of cosmetics on emotions using autonomous responses.	*N* = 60	U.S.	36.7	F	Randomized controlled trial (RCT)	6/11
Querleux et al. (1999)	Study the effects of tactile stimulation on the brain.	*N* = 21	France	Not specified	F	Controlled study	5/11
Roso et al. (2023)	Differentiating two skincare products in terms of appreciation.	*N* = 15	France	(18–65 years)	F	Controlled study	7/11
Sakai et al. (2020)	Evaluate the relationship between product application method and physiological changes.	*N* = 39	Japan	(31–49 years)	F	Not specified	6/11
Sakazaki et al. (2009)	Effects of a cream on the wellbeing of the elderly.	*N* = 11	Japan	73	F	Controlled study	4/11
Sgoifo et al. (2021)	Effects of a cosmetics routine on stress.	*N* = 40	Italy	(25–50 years)	F	Randomized controlled trial (RCT) + single-blind trial	9/11
Springer et al. (2022)	Evaluate the effects of an active ingredient on stress.	*N* = 25	Germany	25.7	F	Single-blind + pseudo-randomized controlled trial	7/11
Tanida et al. (2017)	Evaluate preferences between two lipsticks.	*N* = 14	Japan	22.7 ± 1.6	F	Not specified	5/11
Tanida et al. (2008)	Effect of fragrance on prefrontal activity and stress.	*N* = 31	Japan	22.2 ± 2.3	F	Controlled trial	8/11
Taomoto et al. (2021)	effects of make-up on blind people.	*N* = 16	Japan	Not specified	F	Controlled trial	7/11
Wang et al. (2024)	Compare emotional responses elicited by four cosmetic products.	*N* = 31	China	29.7 ± 4.5	M/F	Randomized controlled trial (RCT)	8/11

**Table 2:** Representation of cosmetics features.

**References**	**Cosmetics used**	**Product brand**	**Application area**	**Place**	**Number of applications**	**Duration of stimulation**	**Amount applied**	**Mode of application**	**Sensory modality**
Abriat et al. (2007)	Five scented products	Not specified	Face	At home	Five applications (every morning for 5 days)	Free	Free	Self-application	Olfaction
Baer et al. (2018)	Nine luxury perfumes and nine non-luxury perfumes	Thierry Mugler, Chanel, Calvin Klein, Kenzo, Dior, Dolce and Gabbana, Ralph Lauren, Guerlain, and Lancôme	–	Lab	One time per product	2 s per product	–	–	Olfaction + Vision (product name, brand, and packaging)
Barkat et al. (2003)	Nine lipsticks + nine nail varnish vials (five colors, two odors)	L'Oréal	Visualization of actual product	Lab	One time per product	Odor: 1 s every 45 s; Color: 5 s every minute	–	–	Vision (color) + olfaction
Boucsein et al. (2002)	Tree hair samples (untreated, treated with standard shampoo and treated with special shampoo) + 3 videos of hair	Not specified	Touching the product	Lab	One time per product	30 s for hair product, 40 s for videos	–	–	Tactile property
Bouhout et al. (2023)	Facial care (emulsion, mask, serum, cream, and steel cosmetic spoon)	Not specified	Face	Lab	One time per product	1 h	Not specified	By a beautician	The entire product (without odor)
Cabannes et al. (2019)	Two foundations (with or without pigmentary agent)	Not specified	Face	At home	Five applications (over five consecutive days)	Free	Free	Self-application	The entire product (Composition)
Churchill and Behan (2010)	40 perfumes	Not specified	–	Lab	Two times per product	Not specified	–	–	Olfaction
David et al. (2019)	Two facial creams (odor and no odor)	Elizabeth Arden	Face	Lab	One time per product	Not specified	2 ml	Self-application	The entire product (mostly odor)
Field et al. (2005)	Shower gel scented with lavender	Colgate-Palmolive	–	Lab	One time per product	2 min per product	–	–	Olfaction
Gabriel et al. (2021)	Two creams	Not specified	Hand	Lab	Three times per product	1 min per product	Not specified	Self-application	The entire product
Hirabayashi et al. (2021)	Six lipsticks (two different colors: favorite and least appreciated, in three quality levels: high, medium, and low)	MAQuillAGE	Half lips	Lab	One time per product	30 s per product	Free	Self-application	The entire product (quality) and vision (color)
Kawabata Duncan et al. (2019)	Seven foundations (three low quality, three high quality, and one intermediate)	Not specified	Half face	Lab	One time per product	30 s per product	Free	Self application	The entire product (quality)
Kikuchi et al. (2021)	Three kinds of face serums	Not specified	Back of the hand	Lab	Four visualizations per product + four visualizations and applications per product	30 s per condition (attached and unattached cosmetic)	0.2 mL per task block	By a beautician	Vision (packaging) + the entire product
Kim et al. (2022)	Four creams without perfume	Not specified	Left forearm	Lab	Two times per product	30 s per product	Not specified	Self-application with specific instructions	The entire product
Kokubo and Kawano (2016)	Two essences with platinum and without (different color)	Inovex Co. Ltd	Face	Lab	One time per product	Approximately 1–2 min per product	Free	Self-application	The entire product (Composition and habits)
Leão et al. (2017)	Two moisturizing creams	NaturaBrazil	On the body (excluding the face)	At home	One time each day for 30 days	Free	Free	Self-application	The entire product (with modalities studied one by one)
Leong (2022)	12 oil-in-water emulsifiers	Not specified	Not specified	Lab	Not specified	Not specified	Not specified	Not specified	The entire product
Lombardi (2017)	Three lip balms (which differ by one of the emollients)	Not specified	Lips	Lab	One time per product	6 s per product	Free	Self-application	The entire product (composition)
Nagai et al. (2012)	Facial cream	Not specified	On the back of the left hand	Lab	One time per condition	15 s per condition	Free	Self-application	The entire product (verbal explanation, written explanation, application, smell and touch)
Ohira and Hirao (2015)	Four types of emulsions	Not specified	Product visualization on image	Lab	Nine times per product (six times in combination with another product and three times alone)	8 s per visualization	–	–	Vision (brand influence)
Painchault et al. (2020)	Two shampoos and two serums	Klorane	Shampoo = hair; serum = forearm	Rooms in a hotel	One time per product	Free	Free	Self-application	Olfaction
Pichon et al. (2015)	Nine perfumes	Thierry Mugler, Chanel, Calvin Klein, Kenzo, Dior, Dolce and Gabbana, Ralph Lauren, Guerlain, and Lancôme	–	Lab	One time per product	Not specified	–	–	Olfaction
Pössel et al. (2005)	Pictures of women with or without makeup	Not specified	Viewing pictures	Lab	40 pictures (four with make-up and four without, 32 others from IAPS)	6 s per view	–	–	Vision
Querleux et al. (1999)	The skin of an operator modified or not by a cosmetic product	Not specified	Fingers of the dominant hand	Lab	Not specified	Not specified	Not specified	By a beautician	Texture
Roso et al. (2023)	Two skincares with fixed fragrance (emulsions)	Not specified	On the non-dominant hand	Lab	One time per product	1 min per product	Free	Self-application	The entire product (composition)
Sakai et al. (2020)	Lotion + emulsion + facial cream	Not specified	Face	Lab	One time per product	Free	Free	Self-application	The entire product
Sakazaki et al. (2009)	Foundation created by the author	Not specified	Face	Lab	One time per product	Not specified	Not specified	By a beautician	The entire product
Sgoifo et al. (2021)	Two facial creams (placebo and enriched with essential oils)	Not specified	Face	At home	Two times in first experiment + 4-week self-administration (day 1–28)	3 min per product + application for 28 days	Not specified	Self-application with specific instructions	The entire product (composition)
Springer et al. (2022)	Two facial creams (with or without an active compound)	Not specified	On the cheeks	Lab	1 time per product	1 min per product	Not specified	Self-application	The entire product (composition) + Olfaction
Tanida et al. (2017)	Two lipsticks	Not specified	Face	Lab	One time per product	30 s per product	Not specified	Self-application	The entire product
Tanida et al. (2008)	One perfume	Not specified	–	Lab	Three times per day + room fragrance every day for 4 weeks	Not specified	–	–	Olfaction
Taomoto et al. (2021)	Cosmetics without perfume	Tokiwa	Face	Lab	One time per product	Approximately 15 min to apply makeup	Free	Self-application	The entire product
Wang et al. (2024)	Four creams	Not specified	On the back of the left hand	Lab	One time per product	30 s	100 μg	Self-application (specific gesture)	Olfaction, vision, application and feel after application

**Table 3:** Representation of characteristics related to recorded physiological data.

**References**	**Physiological data**	**Data processing**	**Temporal modality**	**Artifact removal/filter**	**Comparison**
Abriat et al. (2007)	EDA + EMG (zygomatic) + RR	EDA and EMG: maximum amplitude; RR; sniffing duration (SD); sniffing frequency (SF)	During olfaction of product (long term = after 5 days of application)	Not specified	Control group without cosmetic + control fragrance
Baer et al. (2018)	ECG + EDA + EMG (corrugator and zygomatic) + RR	Heart rate; respiration maximum amplitude; skin conductance response (conductance max); square roots to normalize EDA; mean EMG amplitude; average amplitude percentage	During olfaction of odor	ECG = low pass filter: 30 Hz; EDA = low pass filter 1 Hz; EMG = band pass filter 20–400 Hz + low pass filter: 40 Hz	Non-luxury condition and baseline
Barkat et al. (2003)	ECG + EDA + RR	HR in beats per minute (bpm); SC amplitude in micro siemens (μS)	During olfaction and visualization of color	Not specified	A control without color and odor
Boucsein et al. (2002)	ECG + peripherical blood pressure + EDA + EMG (zygomatic major, levator labii superioris, corrugator supercilii)	Frequency of non-specific skin conductance reactions; mean amplitude of all skin conductance reactions; mean pulse volume amplitude; HR, RMSSD and interbit interval (IBI), EMG: summing up the amplitudes	During all touch	Not specified	Sample without treatment
Bouhout et al. (2023)	ECG + RR + EMG (trapezoid muscle) + EEG	Frontal alpha/beta ratio (power spectral density (PSD) alpha (8–13 Hz) divided by the PSD of beta (13–30 Hz); HRV = LF/HF ratio	During 1-h facial skincare	EEG = artifact removal + high pass filter (<0.01 Hz) + low pass filter (>100 Hz); EMG = filtering standard (5–20 Hz and 200 Hz-1 kHz)	Group without facial skincare
Cabannes et al. (2019)	Cortisol	Mean cortisol concentration	Long term (5 days of application) = Cortisol on waking, 30 min after application, 1 h after application, and 1,900 h after application.	–	Placebo
Churchill and Behan (2010)	EEG	All the classical frequency bands (delta: 0–3.5 Hz, theta: 4–7 Hz, alpha: 8–13 Hz, beta 1: 15–30 Hz and beta 2: 31 + Hz)	During olfaction	Not specified	None
David et al. (2019)	fMRI	Amplitude of Low Frequency Fluctuations (ALFF) [and fractional ALFF (f/ALFF)]	After application	Not specified	Cream without perfume
Field et al. (2005)	ECG + EEG	AFA = right and left frontal alpha powers (R-L); HR (bpm); average activity per frequency (delta, theta, alpha, and beta)	Before, during, and after olfaction	EEG: manual artifact removal + high pass filter 1 Hz + low pass 100 Hz	None
Gabriel et al. (2021)	EEG	Valence = AlphaF4—AlphaF3; Arousal = BetaF3 + BetaF4 + BetaAF3 + BetaAF4)/(AlphaF3 + AlphaF4 + AlphaAF3 + AlphaAF4); theta/beta ratio	Real time during cosmetic application	Automatic artifact removal	None
Hirabayashi et al. (2021)	fNIRS	Changes in concentration of oxygenated hemoglobin (oxy-Hb) and deoxygenated hemoglobin (deoxy-Hb) in prefrontal cortex	Before, during, and after application	Artifact removal with an algorithm	Individual = Less liked color
Kawabata Duncan et al. (2019)	fNIRS	Changes in concentration of oxygenated hemoglobin (oxy-Hb) and deoxygenated hemoglobin (deoxy-Hb) in prefrontal cortex	Before, during, and after application	Artifact removal with the minimum wavelet description length (Wavelet-MDL)	Group with low frequency of foundation use
Kikuchi et al. (2021)	fMRI	Blood oxygenation level (BOLD)	During vision and during application+ vision	Not specified	Non-attached cosmetics
Kim et al. (2022)	EEG	Time frequency analysis = Morley wavelet decomposition	Before, during, and after application	Remove 60 Hz noise + high pass filter: 1 Hz	None
Kokubo and Kawano (2016)	EEG	Power spectrum. average power of alpha waves	During all the experiment = during application	Not specified	Essence without colloidal platinum
Leão et al. (2017)	Cortisol	Level of cortisol	Long term = after 15 days, after 30 days (end of the intervention), and at the 30-day follow-up.	–	Cream without perfume + control group (no intervention)
Leong (2022)	EEG	Power of alpha and beta frequency	During application	Artifact removal with EEGLAB software	None
Lombardi (2017)	EEG + EDA	Alpha and beta waves; Peak detection method (number of peaks/min that occur during a condition); EEG + GSR combination = IOP	Before, after, and during all applications	EEG: bandwidth from 0.2 to 45 Hz + automatic rejection + notch filters: 50 Hz and 60 Hz.	None
Nagai et al. (2012)	fNIRS	Temporal changes in concentrations of oxygenated hemoglobin (oxy-Hb), deoxygenated hemoglobin (deoxy-Hb), and total hemoglobin.	During all conditions (verbal explanation, written explanation, application, smell, and touch)	Deleting data with artifacts	Different conditions
Ohira and Hirao (2015)	EDA	Mean SCR amplitudes (μS)	During visualization of product picture	Low-pass filter: 5 Hz	None
Painchault et al. (2020)	ECG + EDA	HR (bpm); IBI; average EDA; mean pulse pressure	Before, during, and after application + during olfaction	not specified	A control shampoo (without fragrance) and a control serum (water)
Pichon et al. (2015)	ECG + EDA + EMG (right frontalis, corrugator, and zygomatic) + RR	Respiration amplitude: skin conductance transformed into square roots to normalize the data; specific skin conductance response; EMG amplitude; EMG score in %; HR = % of BPM	During olfaction	Respiration activity = high pass filter: 0.025 Hz; heart rate = low pass filter: 30 Hz; EMG = band pass from 20 to 400 Hz + low pass filter below 40 Hz.	Odor group
Pössel et al. (2005)	ECG + cortisol + Immunoglobin A	Heart rate; saliva volumes; concentration of cortisol and IgA	FC = during visualization; Cortisol + IgA = before and after visualization	ECG: Bandwidth 0.3–100 Hz.	IAPS pictures
Querleux et al. (1999)	fMRI	Extent and distribution of neural activity in the primary and secondary somatosensory cortex	During all the experiment = touch	Manual artifact removal	Skin without cosmetics
Roso et al. (2023)	EEG	Valence = frontal alpha asymmetry (8–12 Hz); excitation = ratio between beta (12–28 Hz) and alpha (8–12 Hz) band power	Real time during cosmetic application	No method for removing artifacts	Resting brain activity + Products between them
Sakai et al. (2020)	ECG	HF/LF ratio; coefficient component of variance (ccv) of the LF/HF ratio; HF	Before and after application	Not specified	Non-enjoyment group
Sakazaki et al. (2009)	EEG	DIMENSION analysis (dipolarity D; Dα and Dσ) = EEG alpha-component	Before and after application	Not specified	None
Sgoifo et al. (2021)	ECG + cortisol	HR (bpm); HRV parameters: high frequency band (HF; 0.15–0.4 Hz); low frequency (LF; 0.04–0.15 Hz) to high frequency ratio (LF/HF); cortisol level	Real time throughout the applications + long term = after 4 weeks of application at home	Not specified	Cream without essential oil
Springer et al. (2022)	EEG + cortisol + α-amylase	Power spectral densities for the alpha, beta, gamma, delta, and theta; mean of alpha band powers; cortisol concentrations; α-amylase levels	After application	Not specified	Placebo (without active compound)
Tanida et al. (2017)	fNIRS + ECG	Concentrations of oxy-Hb, deoxy-Hb, and total Hb; right and left dominancy of prefrontal cortex = Laterality Index (LI) (LI = leftΔoxy-Hb – rightΔoxy-Hb); HR	Before, during, and after application	Not specified	None
Tanida et al. (2008)	fNIRS + ECG	Concentration of oxy-Hb, deoxy-Hb, and total hemoglobin; laterality index (LI) = asymmetry of prefrontal cortex activity; HR	Before, during, and after stress task (long term = after 4 weeks of olfaction)	Not specified	Control group (no treatment)
Taomoto et al. (2021)	fMRI	Functional brain mapping	Before, during, and after application	Artifact removal with pretreatment	Blind/sighted group + condition without make-up; + application without cosmetics
Wang et al. (2024)	EEG	• Valence = AlphaF4—AlphaF3 • Arousal = BetaF3 + BetaF4 + BetaAF3 + BetaAF4)/(AlphaF3 + AlphaF4 + AlphaAF3 + AlphaAF4)	During olfaction, vision, application and feel after application	Not specified	Each product and each sensory modality

The different neurophysiological measures used in the articles of this review are: EDA, electrodermal activity; EMG, electromyography; RR, respiration (respiratory rate); ECG, electrocardiogram; fMRI, functional magnetic resonance imaging; fNIRS, functional near infrared spectroscopy; EEG, electroencephalography.

**Table 4:** Representation of the main results.

**References**	**Physiological changes/results**	**Distinction between products/ conditions?**	**Main conclusion**
Abriat et al. (2007)	For the test group: decreased SCR; increased zygomatic activity; decreased RR and SF; increased SD	EDA: Yes; EMG: Yes; RR: Yes	Familiarity with the product induced greater reactivity to its scent when presented again. Increased positive emotions and relaxing effects were reflected on physiological responses in subjects familiar with the range.
Baer et al. (2018)	Fragrance = increased wave and zygomatic activity; no variations in physio measurements depending on the context.	EDA: No; RR: No, ECG: No; EMG: No	The use of psychophysiological indicators for subtle measurements of emotional response to stimuli such as scents may be questioned.
Barkat et al. (2003)	SCR: strong increase for brown; decrease for red, orange, and white; and no variation for pink. For odors, the differences are less visible; HR: colors led to an increase.	EDA: Yes; ECG: Yes	HR and SCR variations depended on the nature of the stimulation (odor or color). Odor: SCR decrease, HR increase; Color: SCR increase, HR decrease. Preferred cosmetic product induced a decrease in SCR and an increase in HR.
Boucsein et al. (2002)	SCR: no variation; ECG: no variation; EMG: greater activity of the zygomatic in response to the treated samples for experts only (knowledge of the product)	EDA: No; EMG: Yes, ECG: No	Hair samples are not strong stimuli to elicit psychophysiological responses.
Bouhout et al. (2023)	EEG: Increase in the alpha/beta ratio significantly greater for the “massage” group; ECG, RR, EMG: Decrease in significant ratio greater for the “massage” group;	EEG: yes; ECG: yes; RR: yes; EMG: yes	Higher physiological wellbeing/relaxation for the massage group.
Cabannes et al. (2019)	Cortisol: difference between the groups with lower concentrations for the test foundation compared to the placebo 30 min, 1 h after application and 5 days after.	Cortisol: Yes	The pigment used brings benefits to the foundation tested as well as a reduction in cortisol concentrations.
Churchill and Behan (2010)	EEG: distinction of relaxing scents	EEG: Yes	The EEG makes it possible to globally see relaxing scents in the same way as those identified using questionnaires.
David et al. (2019)	fMRI: scented cream: higher neural activity in areas involved in smell perception (insula), face perception (fusiform gyrus), reward (preoccupied gray nuclei and the caudate) and in the midbrain area related to general arousal.	fRMI: Yes	The affective and cerebral effects of using a rose-scented cosmetic cream is possible via fMRI.
Field et al. (2005)	EEG: increased left alpha frontal asymmetry after inhalation (alpha decrease, beta increase). No delta variation, theta increase after olfaction; FC: decrease during olfaction and increase afterwards.	EEG: Yes; ECG: Yes	Lavender resulted in greater left frontal activation = positive mood indicator as well as a decrease in HR = Relaxation
Gabriel et al. (2021)	EEG: distinction of the two products with one of the creams resulting in more time with a positive valence = positive emotions.	EEG: Yes	EEG is a useful tool to assess consumer's emotions during product application = enabled the preferences of the subjects to be distinguished.
Hirabayashi et al. (2021)	fNIRS: no difference between the products	fNIRS: No	No correlation between willingness to pay and dorsolateral prefrontal cortex activity. Overall, willingness to pay was not affected by color or quality.
Kawabata Duncan et al. (2019)	fNIRS: group analysis: the application of the different foundations did not lead to significantly different brain activity in the dorsolateral prefrontal cortex; Individual z-score analysis: correlation between willingness to pay and dorsolateral prefrontal cortex activity only for the high-frequency usage group. No discrimination between products.	fNIRS: No	The fNIRS could make it possible to discriminate between products on an individual basis.
Kikuchi et al. (2021)	Highlighting significantly activated brain areas for the attached cosmetic compared to the unattached cosmetic.	fMRI: Yes	The left ventral pallidum (VP) is involved in positive rewards and the posterior cingulate cortex (PPC) is involved in attachment to objects and ARE the central regions of cosmetic attachment.
Kim et al. (2022)	Stacked CNN is the one with the highest-ranking score (75.4%)	EEG: Yes	It is possible to generate a subject-independent computer model to classify the emotions evoked by cosmetic creams.
Kokubo and Kawano (2016)	The test sample resulted in greater alpha activity than during the resting phase in habituated subjects only.	EEG: Yes	Colloidal platinum causes an additional relaxation effect to the cosmetic product.
Leão et al. (2017)	Cortisol = only the multisensory group showed a significant reduction	Cortisol: No	The self-care method (mediated by touch, smell, sight, and hearing) does not reduce stress.
Leong (2022)	There is a good alignment of 83% when the emotion responses of EEG were compared with those from RATA.	EEG: Yes	EEG can be a good tool in studying the emotion of consumers while applying skin care products.
Lombardi (2017)	EEG: difference in the excitement caused by each product + difference in three out of four emotional states (excluding meditation); GSR: excitation (arousal) difference between products	EEG: Yes; EDA: Yes, but mostly individual results; IOP: Yes	EEG alone is good but not enough to understand the emotional effect of products. The EEG + GSR combination (IOP) is essential.
Nagai et al. (2012)	fNIRS: brain areas activated: application = frontal and lateral lobes; olfaction = increased activation in the right frontal areas and decreased in the parietal lobes; tactile = disappearance of activation in the left parietal lobe + decrease in the right frontal lobe	fNIRS: Yes	Different ways and contexts of presenting a face cream activated different areas of the brain.
Ohira and Hirao (2015)	SCR: greater amplitude for preferred products.	EDA: Yes	SCR can be used to successfully estimate product preference; Robust results because repeated 1 year later
Painchault et al. (2020)	HR: significant decrease for serum and shampoo (during the olfaction phase for shampoo); IBI: increase in the variation after application of shampoo which was higher for the test shampoo compared to the control. No variation for serum; EDA: decrease during and after application of test shampoo, but no effect of serum on EDA; PA and pulse: no variation for shampoo, modification for serum but neither due to application nor olfaction	ECG: Yes, EDA: No, PA and pulse: No; posture: No	Some measurements indicated that the peony scent of the tests shampoo and serum was associated with a relaxation response; Physiological cues are useful and more convincing tools to demonstrate a relaxation response associated with peony scent.
Pichon et al. (2015)	SCR: increase for unpleasant odors and perfumes; HR: lower reduction for unpleasant odors than for pleasant odors; no modification for perfumes; EMG: stronger expressive activity for odors than for perfumes; increased zygomatic activity for pleasant odors and perfumes and increased corrugator activity for unpleasant perfumes and odors. RR: no variation	EDA: Yes ECG: No; RR: No; EMG: Yes	Physiological measurements made it possible to distinguish odors but not perfumes.
Pössel et al. (2005)	HR: image of woman wearing make-up = significantly lower heart rate but no significant difference in salivary cortisol and IgA levels	ECG: Yes; cortisol: No; IgA: No	Slides with women wearing makeup induce more positive emotions than those with women without makeup.
Querleux et al. (1999)	Skin without cream: activation of primary and secondary somatosensory zones (primary contralateral, secondary contralateral, primary ipsilateral, secondary ipsilateral); skin with cream: activation in the same cortical area but marked increase in the ipsilateral primary cortex	fMRI: Yes	The extent and distribution of neural activity in the primary and secondary somatosensory cortices were altered differently by the different stimuli.
Roso et al. (2023)	The two products generated different valences but identical emotional intensity. A significant difference was found in the emotional valence between the two products with one causing positive emotions and the other negative emotions.	EEG: Yes	It is possible to determine participants' preference for cosmetic products using EEG.
Sakai et al. (2020)	Significant reduction in ccv (LF/HF) after application in both groups but more significantly for the pleasure group + reduction in ccv (HF) in the non-pleasure group	ECG: Yes	The cosmetic application movements of the pleasure group have an inhibitory effect on the sympathetic nervous system while those of the non-pleasure group have an inhibitory effect on the parasympathetic nervous system = regular movement and longer duration can reduce the balance of autonomic nervous system activity
Sakazaki et al. (2009)	Improving appearance with makeup could help older people resist to loss of mental acuity.	–	The DIMENSION analysis showed an increase in brain activity in seven of the 11 women.
Sgoifo et al. (2021)	• Recording session 1 = HR: bradycardic effects for the two creams (HR decrease). However, only the enriched product produced a significant change in HF and in the LF/HF ratio (HF increase and ratio decrease); cortisol: no significant variation for the two creams. • Recording session 2 = HR increase, HF decrease and higher LF/HF ratio for both groups; cortisol: lower cortisol level for the enriched cream group + better resistance to stress.	Cortisol: Yes; ECG: No	A cosmetic routine that blends aromatherapy and conscious massage is a strategy that promotes resilience to stress with acute and long-lasting physiological, neuroendocrine, and psychological effects.
Springer et al. (2022)	Cortisol: decrease after application of the test cream; α-amylase: no variation for the two products; EEG: greater alpha activity at the temporal level after application of the test cream	EEG: Yes, cortisol: Yes, α-amylase: No	The combination of EEG and cortisol before and after exposure to products with and without active ingredients was found to be suitable for documenting the anti-stress effect of this ingredient.
Tanida et al. (2017)	HR: no significant difference between lipstick A and B; fNIRS: dominant activity on the left prefrontal cortex for lipstick A and no variation for B. + LI of lipstick A was greater than that of lipstick B.	fNIRS: Yes; ECG: No	The 2-channel fNIRS can be useful in the evaluation of preferences = the use of lipstick A induced more activity of the left PFC than the use of lipstick B.
Tanida et al. (2008)	Fragrance for 4 weeks = HR: no significant decrease; fNIRS: right-dominant PFC activity was replaced by left-dominant PFC activity.	fNIRS: Yes; ECG: No	Perfume administration reduced the level of sebum secretion by modulating stress-induced PFC activity.
Taomoto et al. (2021)	Nucleus accumbens (reward system): increase in its activity by make-up for blind women compared to sighted women = the joy felt from make-up was stronger for blind women; pallidum = increased activity after applying make-up in blind women but no difference in sighted women; hippocampus = slightly stronger activity in blind women.	fMRI: Yes	Reward system activity was significantly higher in visually-impaired people than in sighted people of the same age. cosmetics are also beneficial for blind people.
Wang et al. (2024)	It was possible to distinguish the cream that generated the most positive emotions. The same was true for the least appreciated cream (more negative emotions).	EEG: Yes	EEG can be used to detect differences in valence during different interactions with cosmetic creams (olfaction, vision, application, and post-application sensation). However, it was difficult to differentiate between products in terms of arousal (intensity of emotions).

The different neurophysiological measures used in the articles of this review are: EDA, electrodermal activity; EMG, electromyography; RR, respiration (respiratory rate); ECG, electrocardiogram; fMRI, functional magnetic resonance imaging; fNIRS, functional near infrared spectroscopy; EEG, electroencephalography.

### 3.1 General cosmetics characteristics

The products studied could be classified into four categories: make-up (foundation, lipstick, varnish), skincare (creams, cosmetic routine, serum, lip balm), fragrance, and others (cosmetic products not attributable to any of the other categories: essence, shower gel, shampoo, and emulsions). The most studied cosmetic products were skincare (*n* = 16), make-up (*n* = 8), other products (*n* = 5), and fragrances (*n* = 4).

These studies examined different sensory modalities. Some analyzed the whole cosmetic product (*n* = 19), others one or more specific modalities (*n* = 15). Olfaction was the most studied sense (*n* = 10). Then vision was used (*n* = 7), with studies on the effects of packaging, cosmetic color, and brand influence. Few studies were carried out on tactile perception (*n* = 2).

### 3.2 Measurement tool characteristics

In the selected articles, 10 types of measurement were used to assess cosmetics-induced emotions. Of these, physiological tools were used 37 times and neurophysiological tools 21 times (some studies using several of these tools).

Of the 33 studies, 21 recorded brain activity (four with fMRI, five with fNIRS, and 12 with EEG); 12 chose cardiac activity parameters (heart rate, LF/HF ratio, HRV); seven chose hormone/protein assays (five with cortisol, one with alpha amylase and 1 with IgA); eight used electrodermal activity; five used EMG to record muscle activity of different muscles (*n* = 5); and five included respiration.

**Figure 2:**
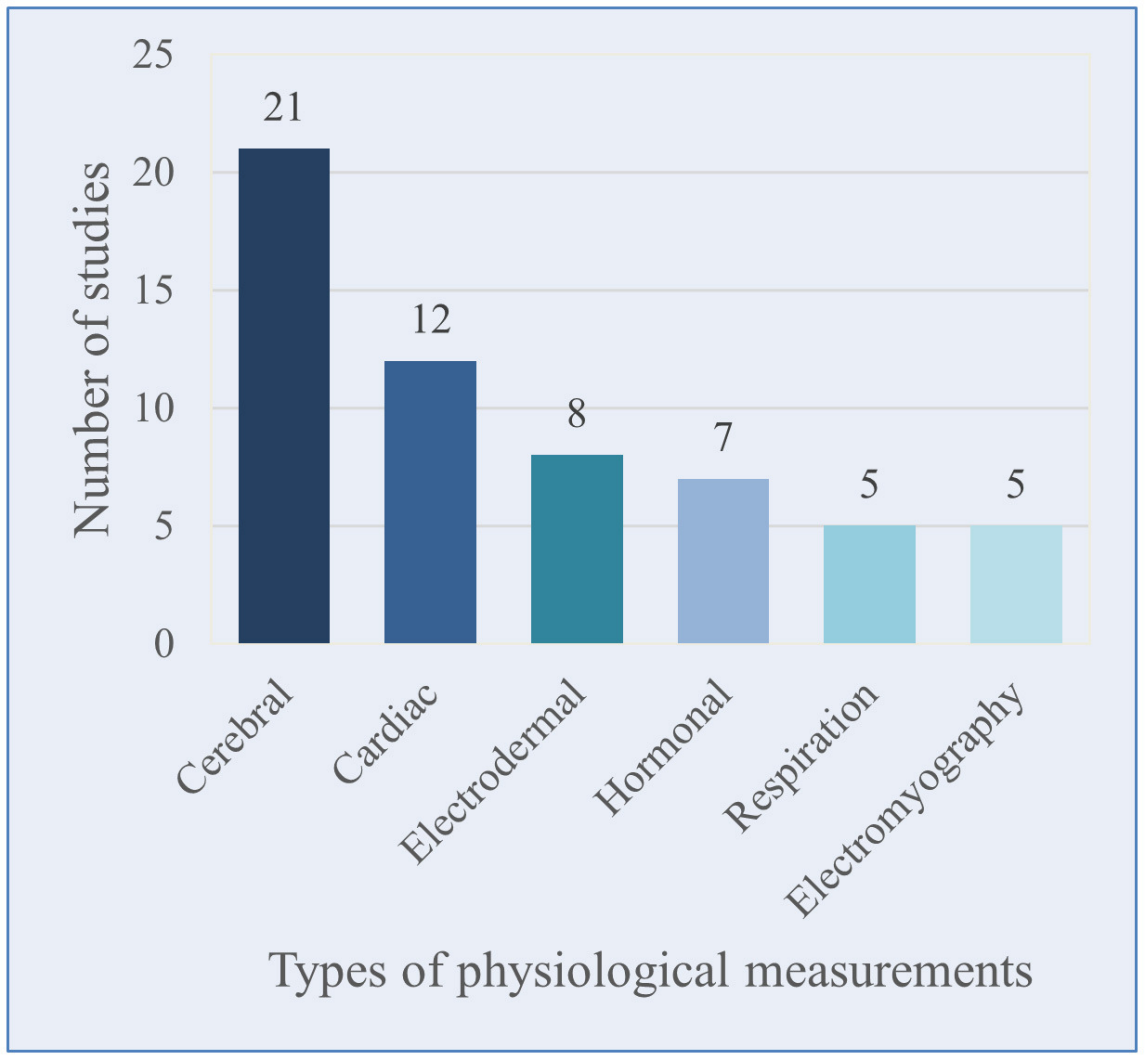
Classification of the studies number according to physiological measurements.

The number of uses applied for each measurement is shown in Figure 2. It is important to note that each study used a different number of measurements. Indeed, the majority used one type of measurement (*n* = 19), while others used two (*n* = 4), three (*n* = 7), or even 4 or more (*n* = 3).

### 3.3 Processing and analysis of recorded data

#### 3.3.1 fMRI

fMRI was used in four studies to observe brain activity resulting in different contexts of cosmetic product presentation. The main technique used was to take cerebral blood oxygenation levels (BOLD) for functional brain mapping in defined areas of interest (*n* = 3). One study added the measurement of low-frequency amplitude fluctuation (*n* = 1). This last technique, known as fALFF, consists in calculating the sum of the amplitudes of specific low-frequency waves characterizing neuronal networks in the resting state. It highlights the most intensely activated areas of the brain.

One of these studies showed that make-up activated different brain areas more intensely in blind women than in sighted women (Taomoto et al., 2021). The areas concerned are the nucleus accumbens, with an increase in activity during the make-up application phase; the pallidum, which has an increase during the phase following application (afterwards); and the hippocampus, whose activity is higher in blind women during make-up application. These brain areas are associated with the reward system, suggesting that visually-impaired women experience greater pleasure when using make-up, even in the absence of visual feedback. A second study sought to determine the brain regions involved in attachment to luxury cosmetics (Kikuchi et al., 2021). The results showed that the left ventral pallidum (VP), involved in positive reward, and the right posterior cingulate cortex (PCC), linked to self-concept, were the central regions for cosmetic attachment. Another work looked at the influence of odors on emotions during the use of cosmetic products (David et al., 2019). When using a scented cream, brain activity in areas involved in smell (insula), face perception (fusiform gyrus), reward (basal ganglia with the caudate nucleus), and general arousal (midbrain area related to general arousal) was higher than when applying a cream without scent. The latest study examined the impact of cosmetic touch on brain activity (Querleux et al., 1999). The application of a cosmetic product altered the skin perception and led to the activation of different sensory areas compared to the touch of skin alone (not having undergone cosmetic treatment). These results suggest that cosmetics modify the integration of tactile information in the brain.

#### 3.3.2 EEG

EEG was used in 12 studies to assess the impact of cosmetics on brain activity. A total of seven different measurements were used. Most studies focused on alpha and beta waves at frontal level (*n* = 7). These studies are intended to calculate emotional valence using frontal alpha asymmetry (*n* = 6) and/or emotional arousal using an alpha/beta wave ratio (*n* = 5). One study used frontal alpha and beta waves to determine four emotional levels (engagement, excitement, frustration, and meditation), but the calculations used were not shared. Other studies focused on the alpha band alone (*n* = 3). In these studies, the analysis focused on different areas, with one study concentrating on the prefrontal and medial parietal electrodes (average power of alpha waves); a second study concentrating on the temporo-parietal level (mean of alpha band power); and the last study focusing on the entire scalp, using a specific method called DIMENSION analysis. This is a method of diagnosing neuronal dysfunction where the distribution of alpha activity is observed in order to verify the stability of brain activity. In addition, one study also used the theta/beta ratio at the medial electrodes (Fz, Cz, and Pz) as a marker of cognitive processing capacity (*n* = 1). Finally, some studies chose to monitor all spectral densities for the whole scalp to see variations at all wavelengths (*n* = 4).

Most studies used alpha activity to measure asymmetry at the prefrontal level. For example, inhalation of a lavender-scented cleansing gel induced a positive mood, reflected by an increase in frontal alpha asymmetry (decrease in alpha activity at the left prefrontal level) (Field et al., 2005). Other authors have developed a method for visualizing participants' emotions in real time, based on frontal alpha asymmetry for emotional valence and alpha/beta ratio for emotional arousal (Gabriel et al., 2021). This technique enabled us to differentiate between two cosmetic creams, the most appreciated of which can be defined as the one that generates the most time with a positive emotional valence (Gabriel et al., 2021; Roso et al., 2023). In addition, emotional valence (alpha waves at the prefrontal level) made it possible to differentiate the appreciation of four different creams during different stages (olfaction, vision, application, sensations after application; Wang et al., 2024). However, it was not possible to distinguish differences in the intensity of emotions (arousal) for these differents creams. The frontal alpha/beta ratio was also used to study the effects of a facial skincare (Bouhout et al., 2023). The results showed an increase in the alpha/beta ratio at the frontal level, more pronounced during the care (42% higher) than after rest. Finally, one study compared the emotions induced by different lipsticks (Lombardi, 2017). The use of EEG (private protocol, but use of alpha and beta waves) was not sufficient to establish the emotional differences induced by the different lipsticks, but the creation of a ratio resulting from the combination of EEG and electrodermal activity made it possible.

Other studies have also used the alpha component, but in such a way as to determine whether there is an increase or decrease in brain activity. For example, one study recorded all the classic frequency bands (alpha, beta, theta, and delta), but found predominant changes during olfaction only in alpha brain activity (Churchill and Behan, 2010). Another work showed relaxation via an increase in alpha power during the application of products containing colloidal platinum only in users accustomed to this cosmetic product (Kokubo and Kawano, 2016). Furthermore, the application of make-up by beauticians led to an increase in neuronal stability (increase in alpha brain activity throughout the scalp) in elderly women (Sakazaki et al., 2009). A final study showed that the application of cosmetic cream induced an increase in alpha activity in temporal brain regions, indicating a state of relaxation (Springer et al., 2022).

Some studies have used new analysis methods. For example, deep learning has been used to assess emotions linked to cosmetic creams (Kim et al., 2022). The results showed a maximum accuracy of 75.4% in classifying emotions using all brain waves over the entire brain surface with the algorithm.

One study used EEG to assess emotions induced by different skin care products (12 emulsifiers) using alpha and beta waves (Leong, 2022). To verify the validity of EEG for measuring emotional responses, the authors compared the results with those of the “Rate-all-that-apply” (RATA) method, a validated technique for assessing the intensity of different emotional attributes using a 10-point scale. Emotional responses showed good alignment (83% similarity) between EEG and RATA.

#### 3.3.3 fNIRS

Functional near-infrared spectroscopy (fNIRS) was used in five studies to explore the links between emotions and brain activity related to cosmetics use. All fNIRS studies analyzed variations in oxyhemoglobin and deoxyhemoglobin concentrations. The area of interest for the emotions analysis was predominantly the prefrontal cortex (*n* = 4), but one study recorded brain activity at the temporal level (*n* = 1). Some calculated total hemoglobin concentrations in the frontal (*n* = 2) or temporal (*n* = 1) cortex. Two studies included in their analyses a laterality index to calculate the asymmetry of brain activity in the prefrontal cortex (*n* = 2). This laterality index indicates whether brain activity is right- or left-dominant (LI = leftΔoxy-Hb - rightΔoxy-Hb).

Most studies have examined willingness-to-pay (WTP), i.e., the maximum amount consumers are ready to spend on a cosmetic product. In these studies, the aim is to understand consumer preferences. In a first study, results showed that activation of the right hemisphere dorsolateral prefrontal cortex was not related to foundation type or usage habits but could predict willingness to pay in frequent users (Kawabata Duncan et al., 2019). A second similar study sought to replicate these results using lipsticks (Hirabayashi et al., 2021). To better understand the influence of liking on brain activity, participants chose their favorite and least favorite lipstick colors. Similar results to the first study were found (correlation between willingness to pay and increased dorsolateral prefrontal cortex activity), but neither product color nor texture influenced brain activity or willingness to pay. It was therefore not possible to detect any consistent differences in brain activity between the different lipsticks. These results suggest that activity in the dorsolateral prefrontal cortex may be more related to processes of personalization or product selection, rather than a biomarker of participants' preferences.

Another study highlighted the activation of specific brain areas according to different ways of considering a cosmetic cream: application, olfaction, and touch (Nagai et al., 2012). During product application, there was strong activation of frontal and lateral lobes, whereas during olfaction the increase was in the right frontal area and a decrease was present in the lateral areas (parietal lobes). In addition, touch resulted in the disappearance of activation in the left parietal lobe and a decrease in activation in the right frontal lobe.

The influence of fragrance has also been evaluated, for example on stress response (Tanida et al., 2008). After 4 weeks of perfume application, prefrontal cortex laterality (PFC) scores decreased, indicating that right-dominant PFC activity shifted to left-dominant activity with the presence of perfume. Another study was able to distinguish differences in liking between two lipsticks using laterality scores (Tanida et al., 2017). One of the products induced a strong increase in oxy-Hb in the left PFC, as well as a greater laterality index compared with the second product. These results indicate left-dominant brain activity and suggest a positive emotional response. Thus, according to the authors, fNIRS can be used to assess the pleasure/displeasure associated with products, and the most appreciated lipstick provoked a more positive emotional response that was visible via greater left frontal activity.

#### 3.3.4 Electrodermal activity

Eight studies used electrodermal activity to examine the intensity of emotions associated with cosmetic products. All these studies analyzed skin conductance levels (*n* = 8), i.e., levels of electrodermal activity averaged by using different procedures. When we feel an emotion, we will have an electrodermal response, which will be greater or lesser depending on the intensity of the emotion. The most frequent analysis consisted in calculating amplitude expressed in μS (*n* = 6), with the calculation of average amplitude (*n* = 4) or maximum amplitude (*n* = 2) during a specific stimulation period (making it possible to obtain the intensity of the emotional response). Other methods involved analyzing the frequency of non-specific skin conductance responses (*n* = 1), the frequency of specific responses (*n* = 1), the number of peaks per minute produced during a specific period (frequency) and transforming these results into a square root to enable data standardization for inter-individual comparisons (*n* = 2). All these methods enable us to obtain emotional responses that are specific to a stimulus.

Physiological differences were observed according to product color and odor (lipstick and nail polish) (Barkat et al., 2003). Overall, skin conductance decreased when subjects saw the color of cosmetic products, but increased significantly when they inhaled them. These results suggest stronger emotional responses to odors than to product colors. These variations were also influenced by individual preferences, with favorite cosmetics leading to a decrease in skin conductance, and less-liked ones to an increase. Other researchers explored the effect of contextual information evoking luxury, such as product name and brand, but no significant changes in electrodermal activity were found (Baer et al., 2018).

Perfumes have also been studied. A study comparing emotions induced by perfumes and odors showed that unpleasant odors elicited stronger skin conductance, and therefore greater emotional response, than pleasant odors and perfumes. However, no correlation could be found between fragrance appreciation and skin conductance (Pichon et al., 2015). The physiological effects of inhaling the fragrance of a cosmetic product after 1 week's daily use were also assessed (Abriat et al., 2007). Familiarity with the fragrance led to a change in electrodermal activity, reflected in a decrease in maximum skin conductance in the test group (daily use before inhalation session) compared with the control group (no daily use, only one inhalation session). Thus, familiarity with a product led to a reduction in electrodermal activity, and hence a reduction in emotional response.

In addition to vision and olfaction, other modalities have also been studied. One study revealed that the application of shampoo led to a decrease in electrodermal activity, while the application of serum did not produce the same effect (Painchault et al., 2020). A study on the influence of different hair treatments on hair-induced tactile sensations concluded, from the lack of results, that hair samples are not strong stimuli to elicit psychophysiological responses such as electrodermal activity (Boucsein et al., 2002). Electrodermal activity was also used to measure preferences between different cosmetic products (four emulsions). Results showed that preferred products elicited higher skin conductance amplitudes (Ohira and Hirao, 2015). Another study suggested that the use of electrodermal activity alone was not sufficient to determine the emotions induced by different lipsticks (Lombardi, 2017).

#### 3.3.5 Cardiac activity

Twelve studies used cardiac activity to investigate emotions associated with cosmetics. Six variables were used. Heart rate (HR) was the most common (*n* = 10) and involves calculating the number of heart beats per minute. This provides information on the emotional state. Indeed, an increase in HR is associated with stress, while a decrease will be associated with relaxation (Agrafioti et al., 2012). The other most frequently used variables were measurements of heart rate variability (*n* = 4), namely the Low Frequency/High Frequency ratio (LF/HF ratio), which indicates whether there is sympathetic or parasympathetic dominance (*n* = 3); the coefficient of variation (ccv) of the LF/HF ratio, which represents the variability of the LF/HF ratio over time (*n* = 1); and the RMSSD (root mean square of Successive Difference), which assesses changes in the heartbeat intervals between each beat (*n* = 1). All these measurements of heart rate variability give indications of the interactions between the sympathetic and parasympathetic nervous systems. For example, the LF/HF ratio is used to visualize the balance between these two systems. A high LF/HF ratio indicates a predominance of sympathetic activity (stress); conversely, a low LF/HF ratio indicates a predominance of parasympathetic activity (calm, relaxation) (Hachenberger et al., 2023; Kop et al., 2011; Shiga et al., 2021). Finally, the other measurements of cardiac activity used were interbeat interval (IBI; *n* = 2) and blood pressure (*n* = 2).

Some studies have focused on product appreciation. By separating subjects into a pleasure group (those who enjoy facial skin care) and a non-pleasure group (those who do not specifically enjoy facial skin care) during the application of a cosmetic routine, it was shown that heart rate variability (LF/HF ratio and ccv of this ratio) decreased significantly for both groups, but more intensely for the pleasure group (Sakai et al., 2020). This result indicates a dominance of parasympathetic activity, which could suggest relaxation. A second study evaluating the effects of liking a cosmetic product (lipstick) on physiological data showed that there was no significant difference in heart rate between the different products (Tanida et al., 2017).

Other studies have focused on specific modalities such as perfume, or on the whole product. A comparison was made between emotions induced by odors (e.g., fruity) and by perfumes (mixtures of odors) (Pichon et al., 2015). Odor-induced heart rate variations were negatively correlated with hedonic scores (decrease of HR in bpm for pleasant odors). However, no significant correlation with perfume hedonicity scores was found for heart rate. In another study, 4 weeks of perfume use resulted in improved resistance to stress, but not detectable via heart rate (Tanida et al., 2008). However, the scent of a lavender gel did reduce heart rate. These results suggest that the scent of a cosmetic can induce relaxation (Field et al., 2005).

A complete facial skincare resulted in relaxation visible on cardiac activity (Bouhout et al., 2023). A 1-h care resulted in greater relaxation, visible via a decrease in the LF/HF ratio that was 13% higher than that recorded for the control group (resting state). Another study showed that variations in heart rate could depend on the nature of the stimulation (Barkat et al., 2003). Indeed, for odors, a general effect with an increase in heart rate was found. Conversely, for color, a decrease in heart rate was observed. What is more, physiological measurements also demonstrated a relaxing effect after the use of various products (shampoo and serum) (Painchault et al., 2020). Both products caused a decrease in heart rate (after application for the serum; during olfaction for the shampoo). Changes in the variation of the inter-beat interval (IBI) with a strong increase occurred during application of scented shampoo and not for odorless shampoo. In addition, there was a decrease in systolic blood pressure and pulse rate after serum olfaction.

In addition, a 1-month longitudinal study showed different results before (first use) and after 1 month of use (Sgoifo et al., 2021). During an initial application of face cream in the laboratory, bradycardiac effects were observed (decrease in heart rate, increase in HF, and decrease in the LF/HF ratio). Following twice-daily application for 28 days (4 weeks), only minor and non-significant effects on heart rate variability were observed. Thus, the first application of a cream seems to induce significant relaxation which, however, is no longer present after 1 month of application, possibly suggesting habituation effects.

Finally, contextual information evoking luxury, such as a product's brand or name, did not lead to any changes in physiological components, including cardiac activity during fragrance olfaction (Baer et al., 2018). The same was true for the tactile perception of different hair samples that had received different treatments (Boucsein et al., 2002). Indeed, touching hair did not lead to any changes in cardiac parameters (HR, IBI, RMSSD, or mean pulse volume amplitude). However, heart rate decreased significantly when viewing photos of women with make-up only (Pössel et al., 2005).

#### 3.2.6 Respiration

Five studies used respiration to assess emotional responses to cosmetics. These used maximum respiration amplitude (*n* = 5), and the frequency and duration of sniffing (*n* = 1). Indeed, breathing speed and amplitude generally vary according to the emotions felt, and these parameters have already been successfully used to analyze and classify different emotional states (Wu et al., 2012).

Several of these studies reported no changes concerning this physiological measurement during viewing and olfaction of a cosmetic product (Barkat et al., 2003) or during perfume olfaction (Baer et al., 2018). Furthermore, no significant effects were reported, and there was no correlation between the appreciation (valence) of a perfume-type stimulus and the respiration measurement (Pichon et al., 2015).

Two studies have reported conclusive results on respiration. In the first, the inhalation of a fragrance led to a reduction in respiration amplitude, as well as in the duration and frequency of sniffing in the familiarization group who had applied cosmetic products (with the scent of the fragrance) for five successive days prior to the olfaction session (Abriat et al., 2007). In the second, a facial skincare led to visible relaxation via respiration, 13% higher than in the control group that received no treatment (Bouhout et al., 2023).

#### 3.3.7 Hormone and protein assays

Six studies used hormone assays to assess stress levels after different cosmetic stimuli. The most common was cortisol levels, mostly expressed as mean concentration (*n* = 5) and rarely as salivary volume (*n* = 1). For the other hormones/proteins (α-amylase and IgA), although they appear in only one study each, they were analyzed using the mean concentration variable (in the same way as for cortisol). All these hormones provide information on emotional states since specific hormones are released in response to emotionally induced physiological reactions. Cortisol, for example, is often studied as it relates to stress responses (James et al., 2023), and there is a small negative association between cortisol levels and wellbeing (de Vries et al., 2022).

The application of two creams produced different results depending on the time of measurement (Sgoifo et al., 2021). On the first day of the study (D1), participants were asked to come to the laboratory to apply two different creams (essential oil-enriched cream and placebo cream). They were then randomly divided into two groups, one of which had to apply the enriched cream and the other the placebo cream twice a day, every day for 1 month. At D1, after a single application, no significant difference was found between the two creams, whereas after 4 weeks of application, cortisol levels were lower and stress resistance greater for the group applying the enriched cream. In another study, a comparison of two foundations applied for 5 days (test product: lipominoacid compound; placebo product), showed that only the test product led to a significant reduction in cortisol levels (Cabannes et al., 2019). Furthermore, when comparing the effects of two facial creams, one containing an active ingredient (test cream) and the other not (placebo cream), it was shown that only the test cream resulted in a significant decrease in salivary cortisol concentration, but no change for α-amylase (Springer et al., 2022). In other studies, evaluation of the effect of different sensory self-care on stress levels showed no significant difference in cortisol levels for the different groups (control group, odorless cream application group, scented cream application group, and multisensory group) (Leão et al., 2017). Furthermore, viewing photos of women wearing cosmetics did not result in any significant changes in cortisol and immunoglobin A levels (Pössel et al., 2005).

#### 3.3.8 Electromyography

Five studies used electromyography to support the results of other physiological measurements. Most studies analyzed the emotional aspect using the activity of facial muscles (*n* = 4), and only one studied another type of muscle (*n* = 1). Thus, the most observed is the zygomatic muscle, which is the muscle of the smile and whose activity increases with positive emotions such as happiness (*n* = 4). The second most analyzed muscle is the corrugator supercilii (*n* = 3). This muscle is located under the eyebrows and, when contracted, pulls the eyebrows downwards. This contraction is associated with negative emotions of dissatisfaction, and its activity decreases when positive emotions are experienced. The last muscles studied were the trapezius muscle, located in the back and used to determine relaxation by relaxing it (*n* = 1); the frontal muscle which, unlike the corrugator, enables the eyebrows to be raised upwards (*n* = 1); and the upper lip elevator muscle (*n* = 1). For the analysis of results, these studies used either EMG amplitude (*n* = 5), or this same amplitude converted into a percentage to standardize it for comparison between groups and individuals (*n* = 2).

One study obtained no significant results from facial expressions during the presentation of different contextual information associated with the luxury of different perfumes (brand, name) (Baer et al., 2018). Only the fragrance of the perfumes led to an increase in zygomatic muscle activity in this study. In another work, when comparing the effects of odors and perfumes, odors elicited much greater expressive activity than perfumes (Pichon et al., 2015). Indeed, the presentation of pleasant odors and perfumes led to an increase in zygomatic muscle activity, which was greater for odors. Conversely, during the presentation of unpleasant odors and perfumes, corrugator muscle activity was greater, and again more intense for odors.

Another study on the tactile perception of hair samples that had received different hair treatments showed changes at the expressive level only in sensory evaluation experts and not in uninitiated subjects. The experts showed greater zygomatic activity in response to the treated samples. The corrugator and upper lip elevator muscles did not show significant results (Boucsein et al., 2002). On the other hand, 1 week's use of pleasant-smelling skin care products (familiarization) led to an increase in zygomatic muscle activity when inhaling the scent of cosmetics (Abriat et al., 2007). Finally, physiological relaxation following a facial treatment, observable via trapezius muscle activity, was 17% higher than that observed in the control group (resting instead of the treatment) (Bouhout et al., 2023).

## 4 Discussion

This systematic review brought together the available literature using neuroimaging and psychophysiological methods to study the emotional responses elicited by cosmetics in healthy adults. The main objective was to judge the relevance of using these different techniques in the field. In this discussion, we summarize the most significant findings. We will then identify the limitations with a view to proposing directions for future research.

### 4.1 Constraints related to sensory modalities in the study of cosmetic products

Applying cosmetics offers a holistic experience involving all our senses, from vision to touch to olfaction. Each of these senses participates in the sensorial experience provided by cosmetics. For example, smell and texture interact to influence the appreciation of cosmetic products and the associated wellbeing (Courrèges et al., 2021). It is therefore essential to understand the influence of each sensory modality in the emotional genesis associated with cosmetic products. To determine whether the neurophysiological methods used allow this multisensory aspect to be considered, the studies were grouped by sensory modality.

#### 4.1.1 Olfactory effects of cosmetic products

Olfaction is the most studied sense in this review. Studies show that it is complex to discriminate between perfumes. Physiological measurements did make it possible to distinguish differences in appreciation between pleasant and unpleasant odors, but not between different pleasant perfumes (Baer et al., 2018; Pichon et al., 2015). Measurements of electrodermal, cardiac, electromyographic activity and respiration failed to distinguish the slightest variation between such closely related products. Only electrophysiological brain activity seemed to allow us to categorize the complex odors that perfumes represent in terms of appreciation (Churchill and Behan, 2010; Tanida et al., 2008).

Other studies have focused on the olfactory effect of skincare products (creams, serums, and cosmetic routines). Some have demonstrated the effects of scent on a subject's relaxation (Abriat et al., 2007; Leão et al., 2017; Painchault et al., 2020). This relaxation was visible via changes in various physiological measurements, depending on the study: heart rate and blood pressure but not electrodermal activity (Painchault et al., 2020); respiration and electrodermal activity but not electromyography (Abriat et al., 2007). Interestingly, familiarization with the fragrance of a cosmetic cream led to greater relaxation and more positive emotions when the cream was reapplied (Abriat et al., 2007). These results suggest that particular attention should be paid when subjects are accustomed to a specific product, since this could lead to an increase in their sensitivity to the product and therefore in their physiological responses. Finally, the study of brain activity has shown that olfaction of a face cream leads to an increase in activity in the right frontal lobes and a decrease in the parietal lobe (Nagai et al., 2012). This pattern of brain activity is linked to positive emotions (Davidson, 2003, 2004; Hugdahl and Davidson, 2003). In addition, EEG made it possible to distinguish between the emotions elicited by olfaction of different creams (Wang et al., 2024). Similarly, fMRI has demonstrated that a scented cream activates different areas than an unscented cream, including regions involved in a positive effect (David et al., 2019).

The latest results on olfaction involved make-up products (lipstick and nail varnish). It was found that variations in heart rate and electrodermal activity depended mainly on the type of stimuli (Barkat et al., 2003). Olfaction, for example, led to an increase in both measurements. A second study has shown that, depending on the type of product (shampoo and serum), odor will have different effects on cardiac and electrodermal activity (Painchault et al., 2020). Finally, neurophysiological measurements (heart rate and brain activity) showed that the lavender scent of a shower gel triggered positive emotions (Field et al., 2005).

All these results on the smell of cosmetic products show that the study of the emotional effects associated with this modality is complex. Firstly, it has been possible to distinguish the emotions induced by olfaction of whole products and perfumes. However, studies on the smell of whole products allowed us to differentiate products according to the valence and emotional arousal they induced; whereas studies on perfumes only allowed us to highlight the type of emotion induced by the perfume studied, but not to differentiate between différents appreciated perfumes. This difference in results between perfumes and cosmetics could be explained by the fact that for whole cosmetic products, smell may not be the only influence in the emotional induction associated with these products. Future studies should therefore pay particular attention to distinguishing the various sensory effects of the products studied (smell, color, texture, etc.). Most, further studies are needed to define differences in perfume appreciation to compare the emotional profiles associated with pleasant perfumes to classify them in terms of appreciation.

Concerning the relevance of the measurements, electrodermal activity gave contradictory results (relaxation distinction: Abriat et al., 2007; no distinction: Painchault et al., 2020), and hormonal and electromyographic (zygomatic) measurements produced inconclusive results. Only measurements of respiration, heart rate, and brain activity (fNIRS, fMRI, and EEG) highlighted these subtle differences in emotional experience. In addition, the measurement of brain activity stood out and could represent an interesting possibility for discriminating between the pleasant odor blends that are perfumes. In aromachology, numerous studies are already using EEG to assess the appreciation of essential oils, for example (for a review, see: Sattayakhom et al., 2023). In this review, only one study used EEG to investigate the olfactory effects of products, and it would be interesting to continue in this direction because the use of EEG could lead to a better understanding of the physiological and emotional effects of different perfumes.

#### 4.1.2 Visual effects of cosmetic products

Studies on the visual effect of cosmetic products have focused on packaging, color, and product appreciation. Two studies focused on the effects of product color. The first showed that color was important in evoking positive emotions and that, in general, color vision led to a reduction in electrodermal and cardiac activity (Barkat et al., 2003). In addition, favorite cosmetics increased electrodermal activity and decreased cardiac activity. The measurement of brain activity (fNIRS), on the other hand, was not modified by the color of the products (lipsticks), although they were individually selected according to the participants' appreciation (Hirabayashi et al., 2021). Similarly, fMRI did not discriminate personal appreciation between the visualization of one's favorite cosmetic serum and that of another product of the same type (Kikuchi et al., 2021). However, visualization of a subject's preferred products elicits greater skin conductance amplitudes (Ohira and Hirao, 2015). An EEG study showed that the vision of appreciated creams led to visible positive emotions (Wang et al., 2024). Lastly, viewing women wearing make-up leads to a decrease in cardiac activity, but no variation for hormone dosages (Pössel et al., 2005).

These studies are not sufficient to conclude on the emotional effects of viewing cosmetic products. Electrodermal activity measurements brought contradictory results (Barkat et al., 2003; Ohira and Hirao, 2015). Similarly, brain measurements (fMRI, fNIRS, and EEG) were not sufficiently informative to determine what happens at an emotional level when a product is glimpsed. Further studies are therefore needed, and it might be interesting in this context to add the measurement of eye tracking. This would make it possible to correlate neurophysiological data recorded with what the subject is looking at, at a precise moment.

#### 4.1.3 Tactile perception of cosmetic products

Few studies have been carried out on tactile perception to investigate the effects of touch associated with the use of cosmetic products. In these studies, physiological data (electrodermal activity, cardiac activity, and cortisol) showed no variation during various touch experiments (Boucsein et al., 2002; Leão et al., 2017). Only brain activity highlighted certain results. Firstly, fMRI showed differences in brain activation during the touch experiment with, on the one hand, identical but more pronounced activity for touch with cosmetic cream compared with touch without cream (Querleux et al., 1999) and, on the other hand, greater activity in the periaqueductal gray matter (an area involved in attachment) during serum application (Kikuchi et al., 2021). In addition, fNIRS was used to show that touching a cosmetic cream led to a decrease in brain activity in the right frontal lobe (Nagai et al., 2012). Finally, EEG showed that all creams evoked positive emotions (greater alpha activity in the right hemisphere) during the application and post-application phases, regardless of their level of appreciation. However, the most appreciated creams generated greater emotional valence (Wang et al., 2024).

These studies are not sufficient to draw conclusions. Few studies have been done on touch, so many aspects have yet to be elucidated. For example, it would be interesting to compare the effects of application by oneself and by others. Similarly, studies have focused on the tactile perception associated with a single product, but no study has yet compared the tactile perception of different products. It would therefore be interesting to compare not only products that are completely different from one another, but also products that differ only in texture (variation in composition).

#### 4.1.4 Overall effects of cosmetics

Studies on cosmetic products do not always focus on specific sensory modalities. Indeed, in the majority, no distinction is made between the different sensory modalities, and the overall effects of product application are observed. Few physiological data have been used to study whole products. Among them, cortisol levels showed both decreases after product application (Cabannes et al., 2019; Leão et al., 2017; Springer et al., 2022), and no variation immediately after application but only after a period of one month of application (Sgoifo et al., 2021). In the latter study, the measurement of cortisol provided an interesting complement to the results, as it was the only measurement that allowed long-term effects to be detected.

In turn, cardiac activity showed a decrease during the application of a facial skincare (Bouhout et al., 2023), a cosmetic routine (Sakai et al., 2020), and the first application of a cream (Sgoifo et al., 2021). While cardiac activity appears to be effective in studying the emotions associated with skincare product use, the application of lipstick-type make-up did not result in any change in the latter (Tanida et al., 2017).

Finally, electrodermal activity has been successfully used to discriminate lip balms (Lombardi, 2017). Similarly, respiration and electromyography were only used once, but were able to show a state of relaxation induced by a facial skincare (Bouhout et al., 2023). In this study, the use of the trapezius muscle was considered very interesting, particularly in the field of cosmetics. It can be used to measure relaxation levels, without having to place any electrodes on the face, leaving it free for the application of cosmetics.

Most studies on whole cosmetics have used brain activity (*n* = 19). This stands out when compared to other measurements and provides relevant information. fMRI has showed that, in general, the application of make-up led to activation of brain areas associated with pleasure (nucleus accumbens, pallidum, and hippocampus), and that this activation was also present in blind women (Taomoto et al., 2021). In addition, the application of serum-like care also activates attachment and positive reward areas (Kikuchi et al., 2021). The use of fNIRS has produced contradictory results. Only one study was able to distinguish at the level of emotions induced by make-up (Tanida et al., 2017) while the others were unable to show any difference (Hirabayashi et al., 2021; Kawabata Duncan et al., 2019; Nagai et al., 2012). Finally, EEG was used to show a state of relaxation during a facial skincare (Bouhout et al., 2023) or during the application of a cream (Kokubo and Kawano, 2016). However, the results for the cream were only found in subjects accustomed to this product. Similarly, it has made it possible to distinguish between different products for the emotional aspect (Gabriel et al., 2021; Leong, 2022; Lombardi, 2017; Springer et al., 2022; Wang et al., 2024). Interestingly, the ability to visualize brain activity in real time to distinguish the valence and emotional arousal of different products has been demonstrated (Gabriel et al., 2021; Roso et al., 2023). It has then been possible to visualize the most appreciated cosmetic directly, as the latter generates more time with positive valence. Similarly, a study succeeded in classifying the emotions induced by a cosmetic cream using an algorithm (Kim et al., 2022). Finally, it has been suggested that make-up can improve neuronal stability in older women, thereby counteracting the deleterious effects of aging (Sakazaki et al., 2009).

All these studies are promising and provide new results that support the idea that cosmetics are the source of positive emotions. The results suggest that whole products are simpler to study than focusing on a specific sensory modality. In addition, skincare products seemed simpler to study than make-up products. Neurophysiological measurements stood out and provided an overview. fMRI showed that the reward system could be one of the areas of interest, due to the activation of areas such as the pallidum, cingulate cortex, even the nucleus accumbens and hippocampus, during the application of different products. EEG and fNIRS showed that emotional valence could be recorded to determine consumers' appreciation and mood toward different cosmetics. Recording brain activity therefore seems a promising way of detecting emotions associated with cosmetics.

#### 4.1.5 Complexities associated with the study of cosmetics

Some subtleties associated with cosmetic products mean that the use of neurophysiological measurements is complex. Firstly, the emotions experienced during product usage are likely to evolve over time. However, current studies have mainly focused on the product application phase. Yet the emotions felt between the beginning and end of application are likely to vary. What is more, the stages preceding application are likely to generate specific responses (discovery of the product, opening the box, handling, etc.). For example, it might be interesting to study the moment of product discovery to see what emotions are triggered at the precise moment when the subject sees the product. The temporal dynamic is important because, on the one hand, it has been shown that positive emotions measured in the prefrontal cortex vary over the short term and can be observed on time scales in the order of 1 s (Wutzl et al., 2023). Moreover, physiological measurements (skin conductance and cardiac activity) could vary considerably between the first and second exposure to the same stimulus (Emilee and Shashi, 2019).

Furthermore, the number of stimuli provoked by cosmetics (whether in terms of the sensory modalities stimulated or the type of product used) renders the endeavor of comprehending product use quite challenging. As seen above, it seems simpler to study the overall influence of cosmetic products rather than that linked to a specific modality. Similarly, skincare products have seemed less complex to study than make-up or perfumes. Only with the convergence of other results will it be possible to move forward on these issues. In this way, the problem opens a wide range of possibilities for future studies. It is also important to specify that emotions are likely to be different when applying cosmetic products, compared to olfaction or vision, since they are self-induced as you are applying the product to yourself.

Secondly, there are several subtleties linked to the various muscular artifacts caused by the application of the product. In studies, different methods have been used to apply cosmetics. In most cases, it was the participant who self-applied the product, while in others application was done by a professional. As mentioned above, applying a product will involve motor movements on the part of the participants, which will lead to artifacts in the recorded data. Few studies provide information on the methods used to exclude these artifacts from the analysis. It would therefore be important to agree on the use of specific methods, not only for the reproducibility of analyses, but also to ensure that physiological measurements have a good signal quality, without having too much signal removed. For example, new methods for artifact suppression are available and it would be interesting to test them in the cosmetics field because of the importance of taking the motor aspect into account (Ehinger and Dimigen, 2019).

Finally, participants' expectations and motivation are rarely considered. Indeed, these factors can significantly influence emotional responses, as participants' prior beliefs and levels of engagement can modify their experience and influence stimulus processing and may affect how they perceive and react to stimuli, such as cosmetics (Barbalat et al., 2013; Piedimonte et al., 2020). Furthermore, evidence indicates that individual differences impact the brain dynamics associated with large-scale brain networks involved in the regulation of emotions (Deodato et al., 2024; Tobia et al., 2017). Thus, future studies should account for such individual differences to better capture the complexity of emotional responses elicited by cosmetics. In this review, only a few articles have separated groups according to appreciation criteria. One study compared each participant's emotional reaction to their favorite and least favorite products (Hirabayashi et al., 2021) and, in another, groups were divided according to appreciation in the use of specific products (Sakai et al., 2020). Groups of participants were also divided according to their frequency of use toward certain products, with the aim of comparing groups with low frequency of use with groups with high frequency of use (Kawabata Duncan et al., 2019). These studies are of great importance because they pave the way for individual differences to be taken into account. What is more, subjects who were accustomed to a product or felt pleasure on application were often those in whom positive emotions were most visible and intense. In the future, it would be interesting to draw inspiration from these studies, which considered the participant's tastes to discover the emotional specifics linked to cosmetics.

### 4.2 Relevance of neurophysiological measurements

#### 4.2.1 Brain activity

Measuring brain activity seems to be the most promising way of studying the emotions associated with cosmetic use. We must remain vigilant, however, because this measurement may have stood out since it is the one most used by the studies in this review. In this review, studies revealed links between cosmetic application and activation of areas of the reward system such as the pallidum, cingulate cortex, or even the nucleus accumbens and hippocampus (fMRI). In addition, they highlighted the possibility of measuring appreciation for a product and discriminating between different products according to the emotional valence they induce, using the distribution of brain activity between the two cerebral hemispheres (fNIRS: laterality index; EEG: asymmetry).

In the studies in this review, the analysis of brain activity focused mainly on frontal areas. In neuroscience, this is an area widely studied for its known implications in the generation and regulation of emotions. Indeed, neurons in the prefrontal cortex communicate with brain regions involved in emotional processes, such as the amygdala, insula, and cingulate cortex (Can et al., 2023). The neuronal activity of these regions can thus be indirectly recorded. More specifically, frontal asymmetry is most often used to study emotions. Indeed, numerous correlations have been found between frontal asymmetry and subjective wellbeing, showing an increase in frontal alpha asymmetry as wellbeing or positive emotions increase (Urry et al., 2004; Wutzl et al., 2023). However, some studies have had contradictory results, finding no significant association between alpha asymmetry and positive emotions or wellbeing (Cannard et al., 2021; Chilver et al., 2020), and have obtained results on other frequency bands and on other brain areas than the frontal (temporoparietal areas). One of the studies highlighted the importance of alpha asymmetry at the temporoparietal level, indicating a specificity of alpha waves in positive emotions (Cannard et al., 2021), and another highlighted that, in the experience of positive emotions, there was an increase in alpha and delta waves but also a decrease in beta waves (Chilver et al., 2020). In fNIRS too, the ability of fNIRS to detect positive emotions is questioned due to current findings that are inconsistent and contradictory, despite most studies showing an increase in prefrontal activity during the manifestation of positive emotions (Westgarth et al., 2021). In general, studies using brain activity have yet to agree on the neural correlates associated with positive emotions or wellbeing (de Vries et al., 2023). Thus, the cosmetics field is not the only one where the use of brain measurements leads to contradictory results.

In addition to studying the positive or negative aspect of an emotion, it is important to be able to study its intensity. In EEG, for example, it has been shown that arousal can be measured at the frontal level using a ratio between alpha and beta waves (Ramirez et al., 2015). However, this technique is not as widespread as the measurement of emotional valence. To our knowledge there is no technique for measuring arousal using fNIRS. Thus, additional studies are necessary to verify the reliability of these neural biomarkers associated with valence and emotional arousal.

To go further and gain a better understanding, several biases could be avoided. Firstly, studies could focus on other brain waves, to see how reproducible the results are, and whether they validate the involvement of alpha waves. In addition, it would be interesting to focus on all brain areas (and not just the frontal ones), to confirm or not the specificity of this area in emotional interactions. It would also be interesting to study the influence of aperiodic activity (the brain's excitation/inhibition balance), since all studies focus on the periodic signal (Deodato and Melcher, 2023; Jacob et al., 2021; Turri et al., 2023). Indeed, incorporating both periodic and aperiodic components of the brain signal may provide deeper insights into neural mechanisms beyond traditional oscillatory activity, and could help clarify whether specific brain states are consistent across studies. Finally, the use of other recording techniques could help advance studies in this field. For example, one work proposes the coupling of EEG and fNIRS to more accurately measure and improve the analysis of emotions (Sun et al., 2020).

#### 4.2.2 Physiological activity

In addition to brain activity, numerous studies have used various physiological measurements to highlight the effects of cosmetics on emotions. Cardiac activity was the most common. In the studies in this review, it proved promising for studying products in their entirety, but did not seem sufficient to discriminate subtle information such as smell or touch. Furthermore, we found that the most promising variable seemed to be the LF/HF ratio offering the possibility of discriminating between different products, even if they are relatively similar products. This measurement emerged as particularly noteworthy as a gauge of positive emotions. In the field of emotions in general, heart rate and heart rate variability (LF/HF ratio) were both positively associated with positive affect, whereas other cardiac measurements were not (Hachenberger et al., 2023). Similarly, during positive emotions of happiness, there was a decrease in heart rate and high frequencies (Shi et al., 2017). It was demonstrated that the LF/HF ratio could represent an objective measurement of positive emotional states (Shiga et al., 2021). What is more, according to a study aimed at analyzing emotional responses (Pinto et al., 2020), cardiac activity was the most effective activity for classifying emotions. However, the combination of several measurements (cardiac activity, electrodermal activity, and electromyogram) enabled better discrimination.

In addition to cardiac activity, electrodermal activity, respiration, hormone assays, and electromyography were also used by the studies in this review. Here, electrodermal activity proved relevant for discriminating between different products on modalities evoking strong emotions (product in its entirety, color, and odor), although it was less effective when faced with complex stimuli that were difficult to distinguish (information evoking luxury, hair treatments, and products with subtle nuances such as lipsticks and perfumes). Generally, studies on emotion recognition often combine several physiological signals to obtain a more comprehensive view of emotional states. A broad review highlights the fact that combining data from several different measurements corresponds to the most promising method for studying emotions (Balters and Steinert, 2017). Indeed, this will bring several advantages, and the information obtained will be mutually complementary. For example, the analysis of facial expressions will make it possible to visualize emotional valence (Ceccacci et al., 2023), while the use of electrodermal activity will make it possible to measure emotional arousal in the face of rapidly changing emotional states (Jukiewicz et al., 2021). Therefore, even though no correlation has yet been made and results are contradictory concerning these various measurements, further studies are needed.

### 4.3 Limits

This review has several limitations. Firstly, the search equation may have restricted the results. Some articles may have been missed for various reasons, such as the absence of specific keywords in the search equation, or the fact that these keywords were searched for in the abstract section. Secondly, the database used may have led to bias, as some articles may not have been listed. In addition, some articles were not taken into account because they were either written in a language other than English or French (Japanese, Chinese), or the authors had only published the abstract of the article. A third limitation relates to the article selection phase. Some articles led to discussions between the two reviewers. They made decisions based on their subjective opinions. A final limitation comes from the quality of the studies in this review. Indeed, according to the study quality assessment tool (NHLBI, 2023), the majority of studies included in this review are of low (*n* = 2) or medium (*n* = 28) quality, while only a few are of very good quality (*n* = 3) (see Table 1).

Indeed, the samples of participants in the studies are relatively small. The lack of studies is also at the root of the absence of replication of results. Moreover, the wide variations in experimental protocols make it difficult to generalize results (pre-treatment methods, use of different combinations of neurophysiological measurements or variables used for analysis, area and number of stimuli with the cosmetic product studied, time of data recording, points of comparison used, etc.). This high variability in the methodologies used has complicated the comparison of results. Another limitation is the privatization of certain protocols. Indeed, cosmetics is a research field focused on business and prestige, which means that some authors do not share their protocols. Finally, the studies included in this review focused on specific samples composed mainly of women, which limits the generalizability of results for the population. Because of all these limitations, conclusions must be drawn with caution.

### 4.4 New guidelines

Considering the results obtained and the advances made in our understanding of emotional responses to cosmetic products, it is appropriate to propose new guidelines for future research in this field.

First, it seems important to use multidisciplinary integration. Indeed, the results of this review show that it is difficult to accurately detect emotions using a single signal. It is therefore essential to encourage approaches that combine several physiological measurements. Ultimately, it will be necessary to identify not only the most relevant physiological measurements, but also the variables associated with them. To achieve this objective, it is also essential that the authors demonstrate transparency in their results to detect the most relevant variables for each of the measurements.

Rigorous methodologies are also essential. This includes standardized experimental protocols and a sufficiently large sample size. It is then essential to establish reproducible study designs and to agree on different modalities such as recording times (for identical temporal comparisons) or stimulation characteristics (duration, number of repetitions, etc.).

As mentioned above, longitudinal studies are also needed to assess the long-term effects of cosmetic product use on emotional responses. In addition, it would be relevant to compare the first contact with a product with subsequent ones. This would provide a better understanding of how the emotional system adapts to skincare routines.

Another point concerns the use of appropriate comparisons. Whether we are talking about a control group with no specific task and simply at rest, or about cosmetics that differ in one of their sensory aspects (color, smell, or texture), it is important to set them up to gain a finer understanding of the emotional effects of different cosmetics.

Similarly, future studies should increase the ecological validity to get as close as possible to real-life conditions. It is true to say that laboratory conditions do not reflect the real-life conditions of cosmetics users. It is therefore important to try to get as close as possible to home conditions (environment, self-application, and application gestures). In this sense, the emergence of wearable devices (such as mobile EEG or connected watches) should facilitate the carrying out of studies outside the laboratory.

Finally, considering individual variability is a major challenge. Age, gender, and mood can all play a significant role and must be considered in data analysis. The cultural and social influences surrounding cosmetics must also be considered. Moreover, the use of cosmetics is unique to each individual and driven by individual appreciation. Each participant has different preferences (color, smell, product) and reactivity thresholds, which may influence the results. Furthermore, expectations and habits are rarely considered. To date, no correlation has been established between these factors and the expression of emotions associated with cosmetics. It is therefore important to use questionnaires to analyze all this information and to be able to characterize the participants.

By implementing these guidelines, it will be possible to achieve significant advances in understanding the interactions between cosmetic products and our emotions.

## 5 Conclusion

This in-depth review of the use of neurophysiological tools highlights the complex interaction between cosmetics and our emotions, and the importance of considering all the factors involved. The studies in this review provided promising results which correspond to those found in studies using questionnaires, many of which have shown a link between positive emotions and cosmetics (Abriat and Le Garrec, 2021; Kosmala et al., 2019; Rudolph et al., 2019; Zhang et al., 2021, 2020). In addition, certain neurophysiological tools have stood out, providing encouraging results. The recording of brain activity using different techniques seems to provide specific information for all study contexts (sensory modality or whole product). The biomarkers that seem to be emerging concern the frontal areas. However, it is important to conduct supplementary research to better understand the areas and brain waves involved in the emotions associated with cosmetics.

Our review, like others, acknowledges the complexity of emotion studies and discusses behavioral and neurophysiological measures. However, it uniquely focuses on the link between neurophysiological responses and emotions related to cosmetics, an underexplored area. Unlike prior work on general emotional recognition, specific discrete emotion, or specific methods, we address all relevant neurophysiological measures while considering cosmetic-specific factors like sensory modalities and potential biases. This review enriches current knowledge and offers new insights for research in the cosmetics field.

Our understanding of the emotional mechanisms associated with differentiation between cosmetic products is still limited. Indeed, major questions remain unanswered, such as the impact of multisensoriality, which is not always considered in studies. Moreover, one of today's challenges lies in differentiating products with similar valences. The integration of inter-individual differences is also a major issue, as one of the shortcomings of current studies is that the diversity of participants is often insufficient, limiting the generalizability of results. Finally, an important ecological issue is the impact of the environment on emotional experience. Are emotional experiences in the laboratory comparable to or predictable from those in stores or at home?

Thus, to fill these gaps, additional studies are essential to provide a more detailed understanding in this emerging field, where the use of neurophysiological measurements is expanding, but still marginal. This research will have to use several physiological measurements, since each measurements seems to provide specific and complementary data for categorize the emotional responses associated with products. In addition, many parameters will have to be considered. For example, it will be important to consider not only the sensory modalities associated with each product (fragrance, color and texture), but also the individual characteristics of users (preferences, habits, culture). What's more, since this is a relatively recent area of research, a certain number of studies still need to be done. For example, certain sensory modalities are under-represented, such as the tactile modality, which is little studied, or the auditory modality, which is not studies at all. In addition, it would be interesting to study the temporal aspect, by considering the different stages of use, with the evolution over time of the emotions felt throughout the interaction with the product. Finally, only one study used machine learning methods to study emotions. However, these methods are increasingly used in studies of emotional recognition (Zangeneh Soroush et al., 2018a; Al Machot et al., 2019; Pinto et al., 2020), and it would be interesting to extend their use to the field of cosmetics (for reviews of the various emotional recognition systems, see: Zangeneh Soroush et al., 2018b; Arshamian et al., 2022; Egger et al., 2019; Li et al., 2023).

Due to the many complexities related to both measurement methods, but also to the many aspects surrounding cosmetics that can act on emotional induction, this area of research requires experts. Indeed, as we have seen, numerous biases can be present in the analyses, and solid knowledge is necessary to better understand the mechanisms involved in the variation of the measurements taken.

Thus, this review calls for a more holistic approach, with the integration of neurophysiological measurements to obtain a new and more complete vision of emotional responses, as well as considering the many specificities associated with cosmetics. So, even if studies are still few in number, they nevertheless open the way to a better understanding of the emotional effects of cosmetics and encourage continued research.

## Data Availability

The original contributions presented in the study are included in the article/Supplementary material, further inquiries can be directed to the corresponding author.
