# Supplementary material for: Neurophysiological approaches to exploring emotional responses to cosmetics: a systematic review of the literature
Source: Front Hum Neurosci. 2024 Oct 22;18:1443001. doi: 10.3389/fnhum.2024.1443001 (PMC11534817; doi:10.3389/fnhum.2024.1443001)
Supplement: Supplementary file 1 [file Data_Sheet_1.docx]

Supplementary Material

Neurophysiological Approaches to Exploring Emotional Responses to Cosmetics: A Systematic Review of the Literature

Audrey Diwoux^1,2*^, Damien Gabriel^2,3,4^, Marie-Héloïse Bardel^1^, Youcef Ben Khalifa^1^, Pierre-Édouard Billot^2,4^

^1^Beauty Research and Performance, Innovation Research and Development Department, CHANEL Fragrance and Beauty, Pantin, France

^2^Université de Franche-Comté, UMR 1322 LINC INSERM, F-25000 Besançon, France

^3^Centre d’Investigation Clinique, Inserm, CIC 1431, CHU, 25 000 Besançon, France

^4^Plateforme de Neuroimagerie Fonctionnelle et neuromodulation Neuraxess, Besançon, France

*** Correspondence:**[audrey.diwoux@chanel.com](mailto:audrey.diwoux@chanel.com)

# Supplementary Figures and Tables

**Table S1: Representation of different exclusion criteria.**

| **Reason for exclusion** | **Category** |
| --- | --- |
| Wrong population (n = 63) | - Pathology (n=29) - Animals (n= 13) - Skin damage (scar, burn, dermatisis...) (n=16) - Child (n=5) |
| Wrong outcome (n = 57) | - Toxicity analysis (n= 15) - Composition analysis (n= 8) - Quality control and/or efficiency (hydratation, wrinkles ...) (n = 4) - Attractiveness and self-image (subjective data) (n = 12) - Face recognition (emotion recognition after makeup or surgery) (n = 14) - Skin properties and aging (n= 4) |
| Wrong induction (n = 273) | - Plastic surgery (n = 56) - Excluding cosmetics (n= 105) - Subcutaneous injection (hyaluronic acid, botox, etc..) (n = 23) - Essential oil (n =35) - Food (n = 17) - Aromatherapy (n= 36) - Nature/city (n=1) |

**Table S2: Definitions of the main measurement tools used by the articles in this review.**

| **Emotional measurement tool** | **Definition** |
| --- | --- |
| Electrodermal activity (EDA) | Measurement of the electrical properties of the skin that are influenced by the activity of the eccrine sweat glands. |
| Electrocardiogram (ECG) | An electrocardiogram is the electrical signals generated by the heart during its beating cycle. |
| Respiration | Respiration refers to respiratory frequency, i.e., the number of complete respiration cycles (inhalation/exhalation) that occur in one minute. It is generally measured in breaths per minute (brpm) or cycles per minute (cpm). |
| Electromyography | Recording of muscular electrical activity to select muscles of interest and observe their solicitation during various activities. |
| Hormone/Protein assays | Hormone and protein assays are commonly used to measure the levels of certain hormones or proteins in blood, urine, saliva or other body fluids. |
| Electroencephalography (EEG) | Electroencephalography is a technique for measuring the brain's electrical activity using electrodes placed on the scalp. EEG provides crucial information on brain function. |
| Functional magnetic resonance imaging (fMRI) | Magnetic resonance imaging is a non-invasive technique that uses the magnetic properties of hydrogen atoms to create detailed images of the human body. This tool enables detailed visualization of the human body's internal structures, particularly soft tissues (brain). |
| Functional Near-infrared spectroscopy (fNIRS) | Near-infrared spectroscopy is a non-invasive technique that measures brain activity by detecting changes in blood oxygenation in the brain. |

**Table S3: Research quality evaluation grid.**

TABLE N°5 : Quality assessment of studies was carried using two instruments developed by methodologists at the National Institute of Health (NHLBI, 2023). The quality assessment tool for observational cohort and cross-sectional studies (QATOCCS) was merged with the quality assessment tool for controlled intervention studies (QACIS). This merger was carried out to have an instrument more specific to the research of this review. It resulted in a total of 11 items for assessing the methodological quality of the different studies (research question, study population, randomization, statistical power, and strength of association between variables). Due to the nature of the physiological studies, two scales were adapted. Firstly, items N°1, 2, 4, 5, 10 and 11 of the QATOCCS have been retained. The other questions were added via the scale for controlled studies (QACIS). From the latter, items 4, 5, 11 and 13 were retained as they were. Items 1 and 2 were then merged (merged into "Was randomization adequate?"). Some questions could not be retained. Items 6 and 12 have not been retained as they were already present in the cohort scale (points 4 and 5 of the latter). Finally, points N° 3, 7, 8, 9, 10 and 14 were eliminated because they were too focused on clinical studies (which is not the case for the studies in this review).

| 1. Was the research question or objective in this paper clearly stated? | Question N°1 QATOCCS |
| --- | --- |
| 2. Has the study population clearly specified and defined? | Question N°2 QATOCCS |
| 3. Were all the subjects selected or recruited from the same or similar populations (including the same time period)? Were inclusion and exclusion criteria for being in the study prespecified and applied uniformly to all participants? | Question N°4 QATOCCS |
| 4. Was a sample size justification, power description, or variance and effect estimates provided? | Question N°5 QATOCSS |
| 5. Was the study described as randomized or a randomized controlled trial? | Question N°1 + 2 QACIS |
| 6. Were study participants blinded to treatment group assignment? | Question N°4 QACIS |
| 7. Were study providers blinded to treatment group assignment? | Question N°4 QACIS |
| 8. Were the outcome measures (dependent variables) clearly defined, valid, reliable, and implemented consistently across all study participants? | Question N°11 QATOCSS |
| 9. Was the exposure(s) assessed more than once over time? | Question N°10 QATOCSS |
| 10. Were outcomes assessed using valid and reliable measures, implemented consistently across all study participants? | Question N°11 QACIS |
| 11. Were outcomes reported or subgroups analyzed prespecified (i.e., identified before analyses were conducted)? | Question N°13 QACIS |

## Supplementary Figures

**Supplementary Figure 1.** The figure legends are required to have the same font as the main text, 12 point normal Times New Roman, single spaced. Please use a single paragraph for each legend and prepare the figures keeping in mind the PDF layout.
